# Taxonomy and Phylogeny of the Aphid Genus *Nippolachnus* Matsumura, 1917, with Synonymy of the Mysterious *Neonippolachnus* Shinji, 1924 (Hemiptera: Aphididae: Lachninae)

**DOI:** 10.3390/insects15030182

**Published:** 2024-03-08

**Authors:** Mariusz Kanturski, Minho Lee, Katarzyna Koszela, Seunghwan Lee

**Affiliations:** 1 Institute of Biology, Biotechnology and Environmental Protection, Faculty of Natural Sciences, University of Silesia in Katowice, Bankowa 9, 40-007 Katowice, Poland; 2 Laboratory of Insect Biosystematics, Department of Agriculture Biotechnology, Seoul National University, Seoul 151-921, Republic of Korea; v2minmin@snu.ac.kr (M.L.); seung@snu.ac.kr (S.L.); 3 Research Institute of Agriculture and Life Sciences, Seoul National University, Seoul 151-921, Republic of Korea; 4 Museum and Institute of Zoology, Polish Academy of Sciences, Wilcza 64, 00-679 Warsaw, Poland; kat.koszela@gmail.com

**Keywords:** aphids, Hemiptera, Tuberolachnini, morphology, new species, new genus, revision, phylogeny, sensilla

## Abstract

**Simple Summary:**

*Nippolachnus* (Aphididae, Lachninae: Tuberolachnini) is a small but interesting aphid genus whose representatives, in contrast to other Tuberolachnini members, differ in morphology and ecological associations. The systematics of the genus is revised through an integrative approach. Morphological examination and phylogenetic analysis resolved seven taxa within *Nippolachnus* and we present seven hitherto unknown morphs. Moreover, one new genus and a new synonymy are proposed. A key to all species is provided, and all taxa are illustrated (including the SEM morphological analyses). These taxonomic changes also affect the relationships of the tribe Tuberolachnini and are discussed within the subfamily Lachninae as a whole.

**Abstract:**

*Nippolachnus* Matsumura, 1917 is a small aphid genus from the tribe Tuberolachnini (Hemiptera: Lachninae) occurring in Southeast Asia. Species from this genus are quite characteristic and stand out among lachnids for their morphology and ecological associations. We have performed a revision and phylogenetic analyses to elucidate the relationships within *Nippolachnus* and other representatives of Tuberolachnini. Here, the taxonomy of the genus is revised based on morphological data to include seven species, three of them newly described: *Nippolachnus chakrabartii* **sp**. **nov**. from India, *Nippolachnus sinensis* **sp**. **nov**. from China, and *Nippolachnus malayaensis* **sp**. **nov**. from Indonesia. *Nippolachnus* appear to be non monophyletic genus and a new genus, *Indolachnus* **gen**. **nov**., is described to accommodate *Nippolachnus himalayensis* (van der Goot, 1917) as *Indolachnus himalayensis* (van der Goot, 1917) **comb**. **nov**. The new genus is a sister group to the remaining *Nippolachnus* species, which created a monophyletic clade. *Neonippolachnus* Shinji, 1924 **syn**. **nov**. is recognised as a synonym of *Nippolachnus*, and *Neonippolachnus betulae* Shinji, 1924 **syn**. **nov**. as a synonym of *Nippolachnus micromeli* Shinji, 1924. For the first time, a scanning electron microscopy study of the sexual generation of *N. piri* Matsumura, 1917 has been performed. Apterous and alate viviparous females of *N. bengalensis* Basu and Hille Ris Lambers, 1968, *N. piri*, and *N. micromeli*, and alate viviparous females of *N. xitianmushanus* Zhang and Zhong, 1982 are re-described and illustrated, as well as apterous and alate viviparous females of *I. himalayensis* **comb**. **nov**. Hitherto unknown morphs of *N. micromeli*, *N. piri*, and *N. xitianmushanus* are described. A lectotype and paralectotypes of N. x*itianmushanus* are designated herein. Notes on distribution and host plants are given, and keys to apterous and alate viviparous females of the genera *Nippolachnus* and *Indolachnus* are also provided.

## 1. Introduction

Lachninae Herrich-Schaeffer, 1854 are among the most interesting aphids due to their diverse abilities to feed on woody and green parts of deciduous and conifer trees and shrubs [1]. They are also one of the most numerous subfamilies within Aphididae, with about 420 valid species [2]. Representatives of this subfamily are characterised by body and appendages densely covered with setae, a short terminal process of the last antennal segment, very low siphunculi on setose sclerites, three rudimentary gonapophyses, and alatae often with pigmented wings [3,4,5,6,7].

Tuberolachnini Oestlund, 1942 are one of the five recognised tribes within the Lachninae [1,8], and five rather poorly known genera have been listed within this group: *Neonippolachnus* Shinji, 1924; *Nippolachnus* Matsumura, 1917, *Pyrolachnus* Basu and Hille Ris Lambers, 1968, *Sinolachnus* Hille Ris Lambers, 1956; and *Tuberolachnus* Mordvilko, 1909, representatives of which are associated with *Betula*, woody Rosaceae, *Elaeagnus*, and *Salix* [9,10]. In the first molecular phylogeny study of Lachninae, Normark [8] pointed out that specimens of *Tuberolachnus salignus* and *Nippolachnus piri* formed an independent, sister clade to Tramini and indicated the presence of more than three tribes within the subfamily. Later, Chen et al. [1] confirmed Normark’s results and added representatives of *Pyrolachnus*, which were resolved as a sister group to *Nippolachnus*. *Neonippolachnus* and *Sinolachnus* (placed previously in Lachnini)—the least known genera—have so far not been included in a molecular phylogenetic study and were arbitrarily transferred by Chen et al. [1] to Tuberolachnini. Although Normark’s [8] and Chen’s et al. [1] results give a good overview on the relationships of tribes within the subfamily, there are still some uncertainties, taxonomical problems, and confusions within some genera and species, in particular for the poorly known taxa [10,11,12,13]. Recently, Kanturski et al. [14] listed several critical differences between *Sinolachnus* and other Tuberolachnini genera and transferred the genus to the tribe Tramini.

Members of the genus *Nippolachnus* are easy to recognise due to the very pale, delicate, and unsclerotised apterous viviparous females and characteristic large rhinaria, abdominal sclerotisation, and hyaline wings in alate viviparous females. They can also be distinguished from other deciduous-feeding Lachninae by their feeding on leaves, never on woody parts. They occur in East and Southeast Asia, mainly in China, India, Japan, Korea, the Russian Far East, and Taiwan [7,10].

*Nippolachnus* was established as a new genus by Matsumura [15] for the type species *N. piri*, collected on *Pyrus lindleyi* (=*Pyrus sinensis*) in Japan. At the same time, van der Goot [16] described *Lachnus himalayensis* from material received from the Indian Museum, collected in Darjeeling. The third species, *N. micromeli* Shinji, 1924, was quite briefly described again from Japan by Shinji [17]. Due to the insufficient description and the lack of type material, that species was synonymised with the type species [4,18,19]. Basu and Hille Ris Lambers [20] described two more species associated with *Eriobotrya* in West Bengal, *N. bengalensis* and *N. eriobotryae*, and the latter was briefly treated as a synonym of *N. himalayensis* [18]. The last species, *N. xitianmushanus*, was described from China [21] and is treated as a synonym of *N. piri* [10,11,19].

Shinji [17], besides the description of *N. micromeli*, also gave a description of a new genus—*Neonippolachnus*—with the type species *N. betulae* collected from *Betula* sp. From the extremely brief description, no information can be extracted beyond the pigmentation of the aphids and similarities to other taxa. After the description, the genus and species were never collected again, despite many years of aphidological tradition and the work of excellent aphidologists in Japan. Furthermore, as no material of Shinji exists, *Neonippolachnus* remains a “ghost taxon”.

Recently, Kanturski et al. [13] restored the species status of *N. micromeli* during the morphological and molecular investigations of *N. piri* complex on different host plants in Japan and Korea. The authors also provided the first SEM morphological study of representatives of viviparous generations of *Nippolachnus* and pointed out that despite the lack of ocular tubercle, a reduced or residual triommatidium can be found in both morphs under the compound eyes. Until then, *Nippolachnus* was treated as a unique genus within Lachninae due to the lack of ocular tubercles and triommatidia [4,7].

As some of the species were described briefly without figures, or the ones presented do not show sufficient differences, we decided to revise the genus to present detailed descriptions and figures of all available morphs of the *Nippolachnus* species. Additionally, we present the first phylogenetic analysis to clarify the relationships within the genus, especially to determine the position of *N. himalayensis* as standing apart from the rest of the species in some important features. We describe the hitherto unknown fundatrix of *N. piri* and *N. micromeli*, apterous viviparous female *N. xitianmushanus*, and representatives of the sexual generation (oviparous females and alate males) of *N. micromeli* and *N. piri*. As a result of detailed morphological comparison, three new species from China, India, and Indonesia are described and illustrated. We discuss the similarities and differences between species as well as the taxonomic status of *Neonippolachnus*.

This paper is part of ongoing research on the revision and phylogeny of the tribe Tuberolachnini.

## 2. Materials and Methods

### 2.1. Light Microscopy and Abbreviations

The material was examined using a Leica DM 3000 LED microscope and photographed with a Leica MC 190 HD camera (University of Silesia in Katowice). Measurements are given in millimetres after [13,14]. The following abbreviations are used: BL—body length (from the anterior border of the head to the end of the cauda); BW—greatest body width across the middle of the abdomen; HW—greatest head width across the compound eyes or triommatidia; ANT—antennae or their lengths; ANT I, II, III, IV, V, VI—antennal segments I, II, III, IV, V, VI or their lengths (ratios between antennal segments are simply given as, e.g., ‘VI:III’); LS—length of longest setae of ANT III; BD III—basal articular diameter of ANT III; BASE—basal part of last antennal segment or its length; PT—processus terminalis of last antennal segment or its length; URS—ultimate rostrum segments (IV + V) or their length; Rs—Radial sector; III FEMORA—hind femur length; III TIBIAE III hind tibia length; FT I—first segment of fore tarsus; MT I—first segment of middle tarsus; HT I—first segment of hind tarsus or its length, HT Ib—basal length of HT I; HT Id—dorsal length of HT I; HT Iv—ventral length of HT I; HT Ii—intersegmental length of HT I; HT II—second segment of hind tarsus or its length; Fx—fundatrix (stem mother), apt. viv. fem.—apterous viviparous female; al. viv. fem.—alate viviparous female; ♂—male; ♀—sexual (oviparous) female.

Depositories of the material studied: NHMUK—Natural History Museum in London, London, UK; DZUS—Zoology Research Team, Faculty of Natural Sciences, University of Silesia in Katowice, Katowice, Poland; IOZ—Institute of Zoology, Chinese Academy of Sciences, Beijing, China; MNHN—Muséum national d’histoire naturelle, Paris, France NIAES—National Institute for Agro-Environmental Science, Kannondai, Tsukuba City, Ibaraki Prefecture, Japan; SNU—Seoul National University, Seoul, Republic of Korea.

Minho Lee made the biological observations and took the photographs of representatives of *N. micromeli* and *N. piri*.

### 2.2. Scanning Electron Microscopy

Specimens for SEM analyses were preserved in 70% ethanol for several days. From ethanol, the specimens were transferred to 6% phosphotungstic acid (PTA) solution in 70% ethanol and left for 24 h. Dehydration was accomplished through an ethanol series of 80%, 90%, 96% and two changes of absolute ethanol for 10 min each. Absolute ethanol dehydrated specimens were treated with chloroform for 24 h. Dehydrated and cleaned samples were dried using the Leica EM CPD 300 auto-critical point dryer (Leica Microsystems, Vienna, Austria). Dry samples were mounted on aluminium stubs with double-sided adhesive carbon tape and sputter-coated with a 30 nm gold layer in a Quorum 150 T ES Plus sputter coater (Quorum Technologies Ltd., Laughton, East Sussex, UK). The specimens were imaged by the Hitachi SU8010 field emission scanning electron microscope FESEM (Hitachi High-Technologies Corporation, Tokyo, Japan) at 7 and 10 kV accelerating voltage with a secondary electron detector (ESD). Final figure processing was done using Photoscape 3.7 (photoscape.org) and IrfanView 64 (irfanview.com).

### 2.3. Phylogenetic Analysis

#### 2.3.1. Taxon Sampling and Outgroup for Phylogenetic Analyses

We used all currently recognised tribes from the subfamily Lachninae in phylogenetic analyses. Since our target group was the genus *Nippolachnus*, we included all described species, three putative new species, and closely related representatives of the genera *Pyrolachnus* and *Tuberolachnus* belonging to the same tribe—Tuberolachnini. Species from the subfamilies Aphidinae, Calaphidinae, and Thelaxinae were used as outgroups.

#### 2.3.2. Morphological Characters

We analysed all eight species belonging to *Nippolachnus* as well as 33 additional species, of which 30 are Lachninae representatives of all known tribes and genera and three species are outgroups. The morphological matrix was constructed in Mesquite v3.5 [22] based on 122 characters (114 morphological and 8 biological) of apterous and alate viviparous females. Unknown character states were coded using ‘?’, and inapplicable states as ‘–’. The list of characters is provided in Supplementary Materials File S1. The nexus file containing the complete character matrix is available as Supplementary Materials File S2.

#### 2.3.3. Phylogenetic Analyses

The morphological matrix for the total number of taxa under study (41) was analysed using Bayesian inference (BI) and maximum likelihood (ML). The data were analysed as a single partition and in the case of BI, the Mkv model [23] and default settings were chosen a priori. Bayesian analysis was performed using MrBayes ver. 3.2.6 [24] running on CIPRES Science Gateway ver. 3.3 [25]. The analysis used four chains (one cold and three heated) and two runs of 10 million generations with default prior settings, except for the temperature, which was set to ‘temp = 0.08’ for better mixing. A script for the combined analysis in MrBayes is given in Supplementary Materials File S3. The convergence of both runs was assessed in Tracer ver. 1.7.1 [26], as well as by the examination of potential scale reduction factor (PSRF) values and average standard deviation of split frequencies in the MrBayes output. Maximum likelihood (ML) analysis was performed using IQ-TREE ver. 2.1.4 [27]. Node support was evaluated by 10 000 ultrafast bootstrap replicates (UFB) [28] (command line: iqtree2 -s matrix.nex -st MORPH -B 10,000 -nt AUTO -bnni). Trees were examined in FigTree ver. 1.4.4 (http://tree.bio.ed.ac.uk/software/figtree/) and later edited and annotated in Adobe Illustrator 2023. Clade support was estimated by BI posterior probability (PP) and ultrafast bootstrap approximation (UFB) in ML. Nodes with PP > 0.80 and UFB > 90 were considered well supported, nodes with PP = 0.70–0.80 and UFB = 85–90 were considered to be weakly supported, and nodes with PP < 0.70 and UFB < 85 were considered unsupported.

## 3. Results

### 3.1. Phylogenetic Analyses

**Figure 1:**
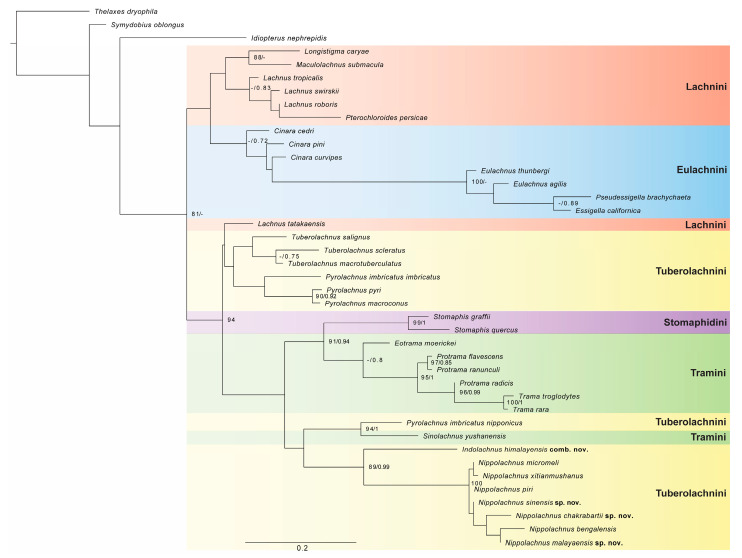
Maximum likelihood phylogenetic tree inferred from morphological dataset. Ultrafast bootstrap (UFB) and posterior probability (PP) support values are shown at the corresponding nodes in UFB/PP format. Tribes of Lachninae are highlighted in colour.

For BI analysis, all independent Markov chains converged on the same stationary distribution as visualised in Tracer, and both combined and individual traces were inspected. The effective sample size (ESS) values were greater than 200 for all parameters, indicating good mixing of the chains. The tree topology presented in Figure 1 is a result of maximum likelihood analysis with support values of both BI and ML.

The topologies of trees obtained from BI and ML analyses are similar for most shallower level clades. However, in the case of the BI tree (Supplementary Materials File S4), many clades were not resolved at a deeper level. Only two (Eulachnini and Stomaphidini) out of five analysed tribes were recovered as monophyletic. However, given the support value for both analyses, their phylogenetic position was not determined. Our targeted tribe Tuberolachnini was resolved in the ML tree in three places. The first clade contains all species of the genus *Tuberolachnus*, sister to three species of the genus *Pyrolachnus*, but this result was unsupported in both analyses. In the ML result, all these taxa were recovered as sister to *Lachnus tatakaensis* Takahashi, 1937 with no support. The second part of Tuberolachnini contains only *Pyrolachnus imbricatus nipponicus*, which in both analyses were resolved as sister to *Sinolachnus yushanensis* from the tribe Tramini with good support (UFB = 94, BI = 1). These two taxa are resolved as sister to the third part of the tribe Tuberolachnini, which contains all representatives of the genus *Nippolachnus*, including three new species. In both analyses, *Nippolachnus himalayensis* was resolved as sister to all other species of this genus with weak and good support (UFB = 89, BI = 0.99). Based on the results of phylogenetic analyses and morphological comparison, we describe three new species in the genus *Nippolachnus—N. chakrabartii* sp. nov., *N. malayaensis* sp. nov., and *N. sinensis* sp. nov.—and decide to move *Nippolachnus himalayensis* to the separate, newly established genus *Indolachnus* gen. nov.

**Figure 2:**
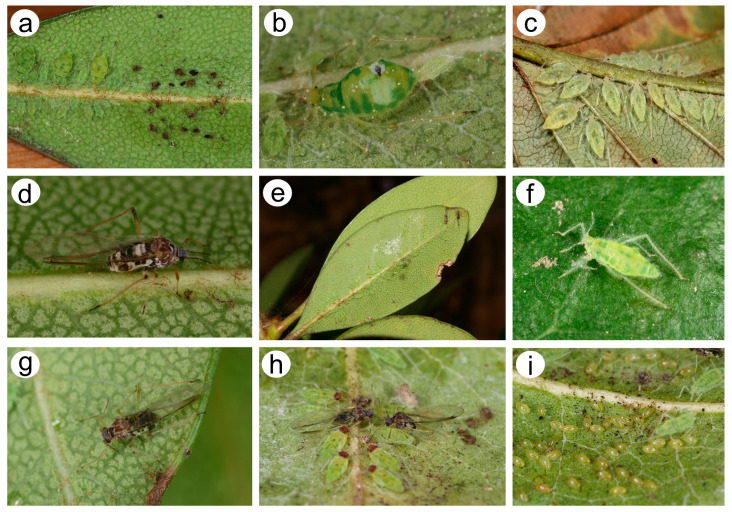
*Nippolachnus micromeli* morphs in life and biology: (**a**) colony of fundatrices and fundatrigeniae nymphs near the egg residues, (**b**) adult fundatrix giving birth to first instar nymph, (**c**) alatoid nymphs, (**d**) alate viviparous female, (**e**) well-camouflaged autumnal colony of sexuales, (**f**) oviparous female, (**g**) male, (**h**) oviparous female and male in copula with male alatoid nymphs, (**i**) oviparous females and overwintering eggs.

**Figure 3:**
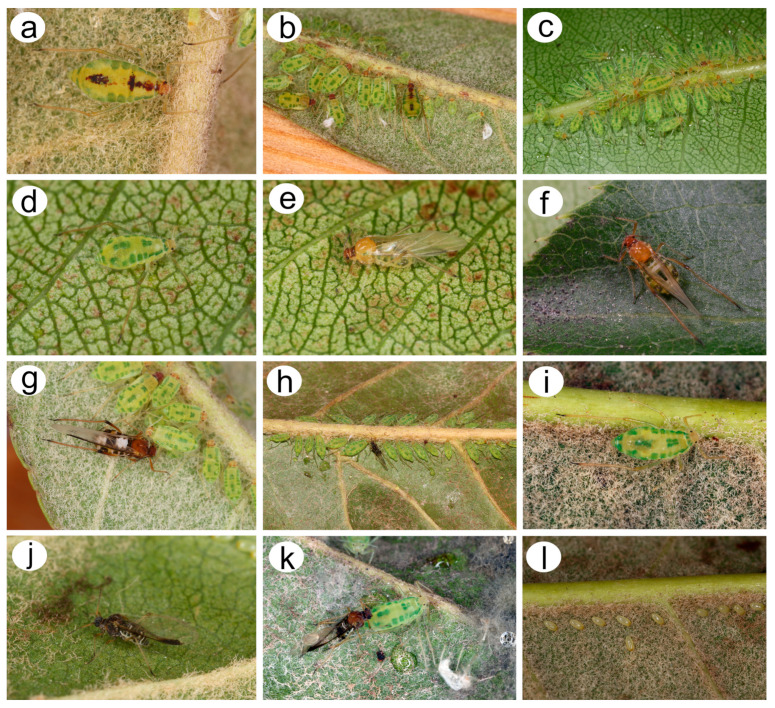
*Nippolachnus piri* morphs in life and biology: (**a**) adult fundatrix, (**b**) colony of fundatrices and alatoid nymphs, (**c**) colony of apterous viviparous females and nymphs, (**d**) apterous viviparous female, (**e**) very freshly moulted alate viviparous female, (**f**) freshly moulted alate viviparous female some time after, (**g**) alate viviparous female final pigmentation during life, (**h**) autumnal colony of sexual morphs and nymphs, (**i**) oviparous female, (**j**) male, (**k**) oviparous female and male in copula, (**l**) overwintering eggs.

**Figure 4:**
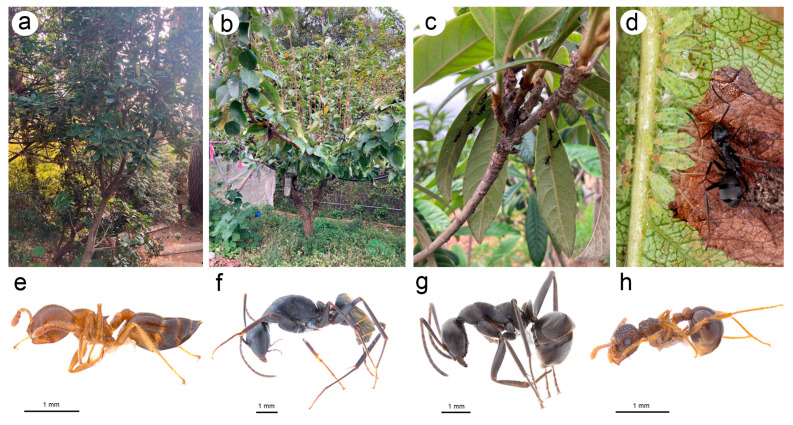
Host plants and attending ants of *N. micromeli* and *N. piri* in Korean Peninsula: (**a**) Raphiolepis indica var. *umbellata*—host plant of *N. micromeli*, (**b**) *Pyrus pyrifolia*—host plant of *N. piri*, (**c**) undersides of *Eriobotrya japonica* with *Camponotus japonicus* attending to indistinct colonies of *N. piri*, (**d**) *Formica japonica* worker with the colony of *N. piri* ready to take the honeydew droplet, (**e**) worker of *Crematogaster matsumurai* attending *N. micromeli*, (**f**) worker of C. japonicus attending *N. piri*, (**g**) worker of *F. japonica* attending *N. piri*, (**h**) worker of *Pristomyrmex punctatus* attending *N. piri*.

### 3.2. Notes of the SEM Morphology of Sexual Generation (Oviparous Female and Alate Male) of Nippolachnus Based on the Type Species N. piri

**Figure 5:**
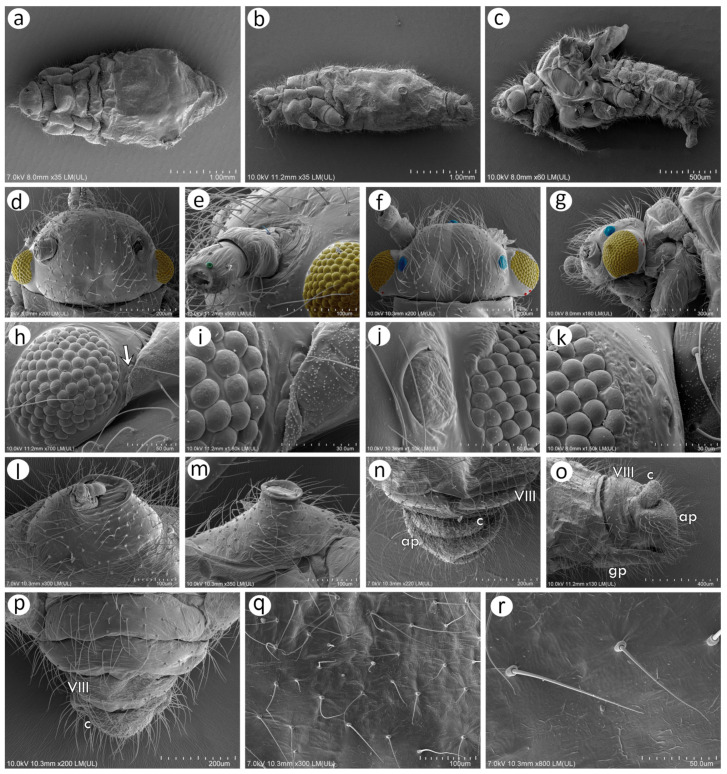
Scanning electron microscopy (SEM) of sexual morphs of *N. piri*: (**a**) dorsal side of the oviparous female, (**b**) lateral side of the oviparous female, (**c**) lateral side of the male, (**d**) dorsal side of the oviparous female head with compound eyes (yellow), (**e**) fragment of lateral side of the oviparous female head with compound eye (yellow) and visible campaniform sensillum on the dorso-lateral side of the antennal pedicel, (**f**) dorsal side of the head of the male with large compound eyes (yellow), three ocelli (blue), and triommatidia (red), (**g**) lateral side of the head of the male with large compound eye (yellow), ocellus (blue), and triommatidia (red), (**h**) compound eye of the oviparous female with hidden triommatidia (arrow), (**i**) ultrastructure of the compound eye ommatidia and triommatidia, (**j**) ultrastructure of the male compound eye ommatidia and ocellus, (**k**) ultrastructure of compound eye ommatidia and triommatidia, (**l**) siphunculus of oviparous female, (**m**) siphunculus of the male, (**n**) dorsal view of the perianal structures of oviparous female showing abdominal segment VIII (VIII), cauda (c), and anal plate (ap), (**o**) lateral side of the perianal area of oviparous female showing abdominal segment VIII (VIII), cauda (c), and anal plate (ap), (**p**) dorsal view of perianal area of the male with abdominal segment VIII (VIII) and cauda (c), (**q**) dorsal cuticle with numerous setae, (**r**) ultrastructure of the dorsal setae.

**Figure 6:**
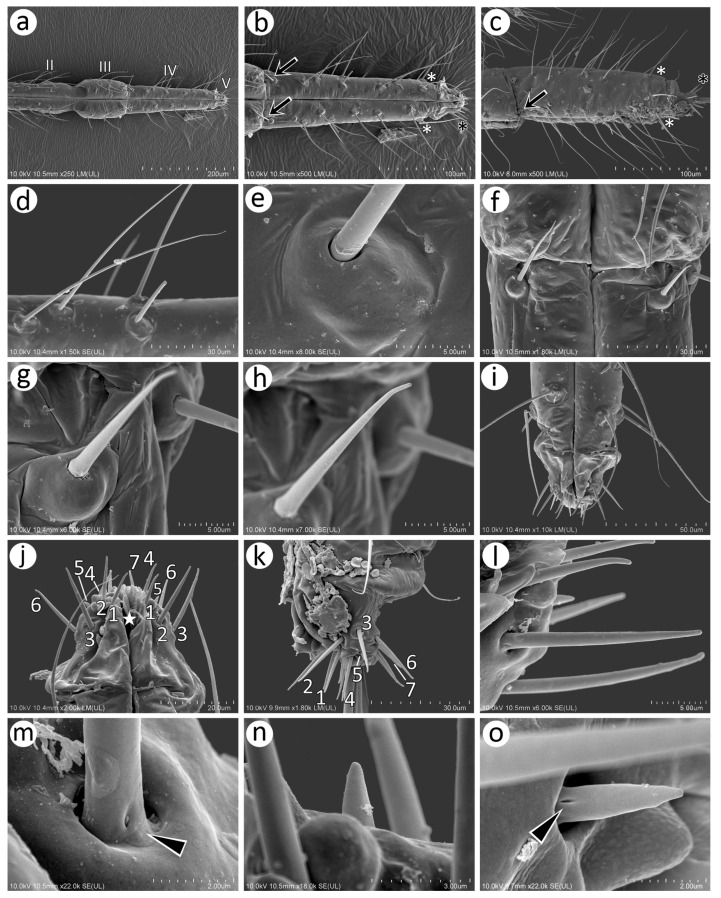
SEM of mouthparts of sexuales of *N. piri*: (**a**) distal part of labium with second (II), third (III), and ultimate rostral segments (IV + V), (**b**) ventral side of URS with numerous trichoid sensilla, of which three distal pairs form primary setae (white asterisks), two type II basiconic sensilla (arrows), and type III basiconic sensilla on the apical part of RV (black asterisk), (**c**) lateral view of the URS showing the above-mentioned sensilla, (**d**) trichoid sensilla on URS, (**e**) ultrastructure of the socket of trichoid sensilla, (**f**) type II basiconic sensilla, (**g**,**h**) ultrastructure of type II basiconic sensilla, (**i**) distal part of RV and RV, (**j**) ventral side of RV with seven pairs of type III basiconic sensilla and stylets opening (star), (**k**) lateral side of RV with seven pairs of type III basiconic sensilla, (**l**) two lengths of type III basiconic sensilla, (**m**) ultrastructure of the basal part of long type III basiconic sensilla with moulting pore (arrowhead), (**n**) ultrastructure of the short type III basiconic sensillum, (**o**) ultrastructure of the basal part of the short type III basiconic sensillum with moulting pore (arrowhead).

As Kanturski et al. [13] performed detailed SEM studies on the viviparous generations (apterous and alate viviparous females) of *N. piri* and *N. micromeli*, here, we focused on the so far unknown sexual generations in this genus—oviparous females and males. In general, sexual generation morphs are similar to the viviparous generations—oviparous females to apterous viviparous females and alate males to alate viviparous females. Oviparae have almost the same shape as the viviparous females, with a slightly enlarged perianal area (Figure 5a,b). The alate male is characterised by a strongly sclerotised thorax and clearly visible genitalia (Figure 5c). In both sexes, the compound eyes are well developed, surrounded by crescent-shaped collars (Figure 5d–f). Triommatidia are present and are lying on the back, outside the compound eyes (Figure 5h,i,k). They are not placed on an ocular tubercle and are much more difficult to find in the oviparous female than in the male (Figure 5g,k). Moreover, the head of the male is characterised by three quite well-developed and large ocelli (Figure 5f,j). Siphunculi are low but well developed and sclerotised, and densely covered with long, fine, pointed setae (Figure 5l,m). The perianal area of the oviparous females is densely covered with long, fine, pointed setae. The anal plate is large, crescent-shaped from the dorsal view, and very flat from the back (Figure 5n,o). In the male, the perianal area is limited to dorsal abdominal segments and visible cauda (Figure 5p), whereas in the oviparous female, the perianal structures are well visible (Figure 5o). The dorsal side of the body of both sexes is densely covered with numerous long, very fine, pointed setae with very well-developed and protuberant sockets (Figure 5q,r). Both sexes have a very similar morphology of mouthparts and sensilla. The labrum is densely covered with long, fine, pointed trichoid sensilla (Figure 6a). The ultimate rostral segments (RIV + RV) are slender and the fifth segment is well separated (Figure 6b). The ultimate rostral segments bear three kinds of sensilla: one pair of type II basiconic sensilla on the ventral proximal part of RIV, trichoid sensilla along the segment, and type III basiconic sensilla on the distal part of the last segment (Figure 6c). The trichoid sensilla are very similar to those on the antennae and other appendages—long, tubular, very fine, especially in the apical part, and pointed (Figure 6d). They have oval, well-developed, and flexible sockets (Figure 6e). Type II basiconic sensilla extend over the border between the segments (Figure 6f), are about 15–20 μm long, have hemispherical and flexible sockets, and their apices are rounded (Figure 6g,h). The distal end of RIV bears three pairs of trichoid sensilla (primary setae) (Figure 6i). The last rostral segment bears seven pairs of long, rigid type III basiconic sensilla, of which the fifth are much shorter than the others (Figure 6j,k). The long type III basiconic sensilla are about 17–18 μm in length, arise from inflexible sockets, and have well-developed moulting pores (Figure 6l,m). The short type III basiconic sensilla are about 3–4 μm long and lie much deeper in the cuticular cavities (Figure 6n,o).

#### 3.2.1. Morphology of Oviparous Female *N. piri*

**Figure 7:**
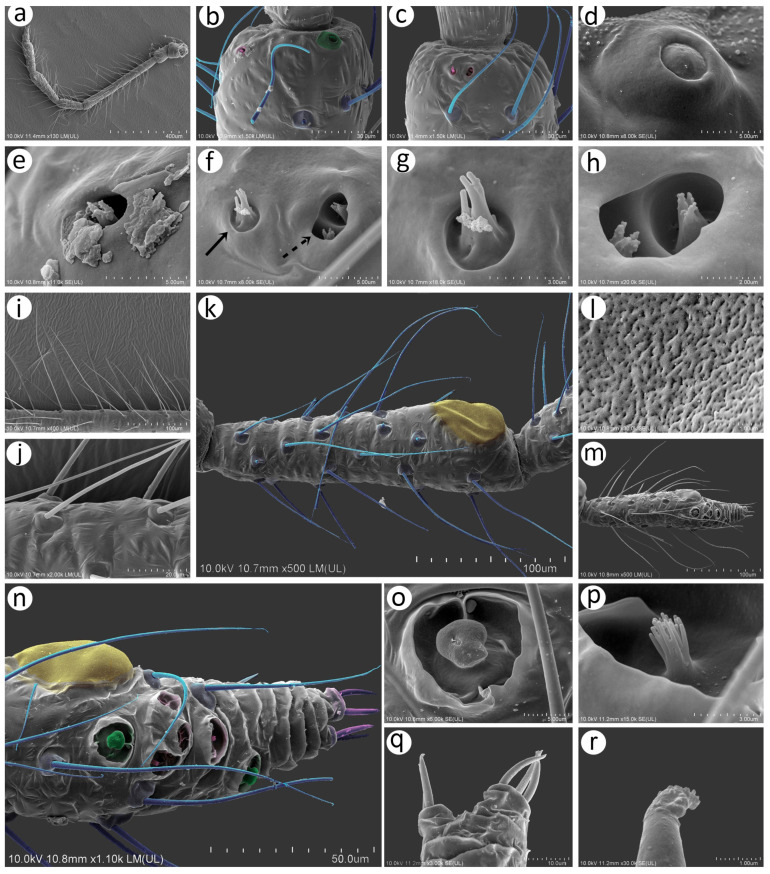
SEM of antennal sensilla of oviparous female *N. piri*: (**a**) antenna general view, (**b**) latero-ventral side of the pedicel showing type I trichoid sensilla (blue), campaniform sensillum (green), and single rhinariolum of type A (pink), (**c**) ventral side of pedicel showing type I trichoid sensilla (blue) and double rhinariolum of two types (pink), (**d**) ultrastructure of the campaniform sensillum, (**e**) ultrastructure of rhinariolum type A, (**f**) group of rhinariola type A (solid arrow) and double rhinariolum type B (dotted arrow), (**g**) ultrastructure of rhinariolum type A, (**h**) ultrastructure of the two rhinariola type B, (**i**) type I trichoid sensilla on antennal segments, (**j**) ultrastructure of sockets and basal parts of type I trichoid sensilla, (**k**) ANT V with type I trichoid sensilla (blue) and large multiporous placoid sensillum (yellow), (**l**) ultrastructure of the porous membrane of the large placoid sensillum on ANT V, (**m**) ANT VI with different kind of sensilla, (**n**) different types of sensilla on ANT VI: type I trichoid sensilla (blue), type II trichoid sensilla (violet), large multiporous placoid sensillum—major rhinarium (orange), accessory rhinaria in form of two small multiporous placoid sensilla (green), and four sunken coeloconic sensilla (pink), (**o**) ultrastructure of the small multiporous placoid sensillum, (**p**) ultrastructure of the type II sunken coeloconic sensillum, (**q**) type II trichoid sensilla on the apical part of the terminal process, (**r**) ultrastructure of the apex of the type II trichoid sensillum.

As mentioned above, the morphology of the oviparous female resembles that of the apterous viviparous female and will be treated separately for some morphological characters together with the alate male (resembling the alate viviparous female’s features). The oviparous female’s antennae are shorter and bear fewer sensilla, of which the majority are characteristically very long trichoid sensilla on all antennal segments (Figure 7a). On the dorsal side of pedicel one, slightly protuberant campaniform sensillum can be found near the segment edge (Figure 7b). The campaniform sensillum is rounded, with both the collar and cap being rounded. The diameter of the sensillum is about 10 μm, and that of the cap about 4 μm; the cap has a small pore, which is not located centrally (Figure 7d). On the ventral side of the pedicel, three rhinariola were found (Figure 7c). Their morphological features differ, and we propose to divide them into two descriptive types: rhinariolum type I and rhinariolum type II. Rhinariolum type I is exposed, lying in a rounded shallow cuticle cavity, about 2.5 μm in diameter (Figure 7f). The type I rhinariolum sense peg is 3–4 μm long, and is divided into 2–3 smooth and longer projections (Figure 7g). The type II rhinariolum, in contrast, lies deep in the cuticle, and is characterised by more (4–5) and shorter projections, which in addition bear spherical protrusions (Figure 7e,f). Additionally, this type of rhinariolum has been observed as a single peg (Figure 7e) and as double sensilla separated by a transverse cuticular diaphragm (Figure 7h). The opening of the single type II rhinariolum is about 2.3 μm in diameter and in the case of the double type, the opening is oval, 4.10–4.20 μm long, and 2.20–2.60 μm wide. As mentioned above, all segments are covered with numerous very long, tubular, fine, pointed type I trichoid sensilla (Figure 7i) with well-developed sockets, which are rounded from the dorsal view and trapezoid from the lateral view (Figure 7j). On ANT V, besides type I trichoid sensilla, one large multiporous placoid sensillum (primary rhinarium) can be noted on the distal part of the segment (Figure 7k). The sensillum is rather flat, slightly oval or rounded, 51–52 μm long, and 27–28 μm wide. The sensillum surface is densely covered with numerous slightly rounded or oval pores, 65–75 per 1 μm^2^ (Figure 7l). The last antennal segment is characterised by the most diverse sensilla (Figure 7m). On the border of the basal part and the terminal process, a large, protuberant multiporous placoid sensillum is present and its apical end designates the beginning of the terminal process. The large, multiporous placoid sensillum (major rhinarium) is about 42–43 μm long and 20–23 μm wide. On the lateral side of the segment (in relation to the side marked by the placoid sensillum), six accessory rhinaria can be found, representing two general types of sensilla. Small placoid sensilla lie in polar positions and one of them lies on the distal part of the base (the rest are moved to the terminal process). Between small multiporous placoid sensilla, four sunken coeloconic sensilla can be found, of which two are of type I and two of type II. The very apical part of the terminal process bears type II trichoid sensilla (Figure 7n). Small multiporous placoid sensilla on ANT VI, which are treated as accessory rhinaria, are mushroom-shaped with an always narrow stem and slightly flat, wide upper part, which is 5–6 μm long and 4.50–5.50 μm wide. They lie in a broad and shallow cuticular cavity and are surrounded by a well-developed cuticular collar (Figure 7o). Type I sunken coeloconic sensilla are exposed and surrounded by a cuticular collar similar to placoid sensilla. They are characterised by numerous (16–18) long projections, some of which have capitate apices (Figure 7p). Type II trichoid sensilla are divided into two groups of three and two (Figure 7q), are rather rigid, 11–12 μm long, and are characterised by scattered apices (Figure 7r).

**Figure 8:**
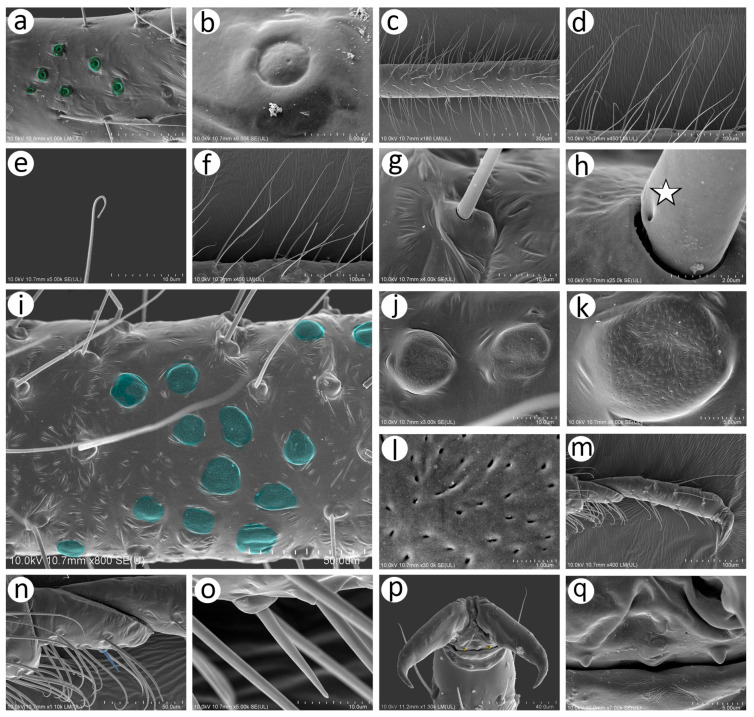
SEM of hind legs of oviparous female *N. piri*: (**a**) inner proximal part of hind femur with campaniform sensilla (green), (**b**) ultrastructure of the campaniform sensillum with visible pore near the middle of the main disc, (**c**,**d**) trichoid sensilla on hind femora, (**e**) ultrastructure of the trichoid sensillum apical part, (**f**) trichoid sensilla on hind tibiae (socket of the trichoid sensillum), (**g,h**) ultrastructure of the basal part of the sensillum with visible moulting pore (star), (**i**) fragment of hind tibia with numerous scent plaques (turquoise), (**j**,**k**) shape and porous surface of the protuberant scent plaques, (**l**) ultrastructure of the porous surface of the scent plaque, (**m**) hind tarsus, (**n**) first segment of hind tarsus chaetotaxy with one peg-like sensillum (blue), (**o**) ultrastructure of the peg-like sensillum, (**p**) claws of second segment of hind tarsus with residual parempodia (yellow), (**q**) residual parempodia.

On the inner side of the hind femora, six campaniform sensilla of two sizes can be found (Figure 8a). The campaniform sensilla on the femora are similar to that on the pedicel but more often their collar is not uniform and a small groove on its lateral side can be noted (Figure 8b). Hind femora and hind tibiae are densely covered with trichoid sensilla, which are also similar to those on the antennal segments (Figure 8c,d,f), but much finer in the distal parts, which makes them curved (Figure 8e). The sensilla are tubular, arise from well-developed and movable sockets with, in some cases, visible moulting pores (Figure 8g,h). The hind tibia of the oviparous females bear clearly visible, rounded or slightly oval scent plaques (Figure 8i,j). The scent plaques are, moreover, slightly protuberant, about 14–15 μm in diameter, and are characterised by clearly visible, rounded to spindle-shaped pores (Figure 8k,l), 5–6 per 1 μm^2^. Hind tarsi are of typical shape with fine, curved, pointed setae (Figure 8m). On the first segment of the hind tarsus, one peg-like sensillum can be found besides much longer and flexible trichoid sensilla (Figure 8n,o). Parempodia are barely visible and residual, in the form of very low cones of rounded apices (Figure 8p,q).

#### 3.2.2. Morphology of Alate male *N. piri*

**Figure 9:**
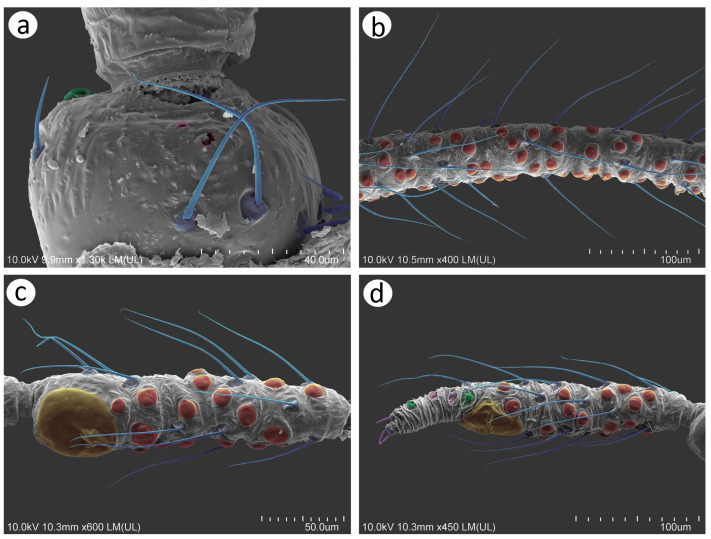
SEM of general view of antennal sensilla of the alate male *N. piri*: (**a**) pedicel, (**b**) ANT III, (**c**) ANT V, (**d**) ANT VI, campaniform sensillum (green), rhinariola (pink), type I trichoid sensilla (blue), small multiporous placoid sensilla—secondary rhinaria (orange), large multiporous placoid sensilla (major rhinarium on ANT VI) (yellow), small placoid sensilla (accessory rhinaria on ANT VI) (green), sunken coeloconic sensilla (accessory rhinaria) (pink), type II trichoid sensilla (violet).

**Figure 10:**
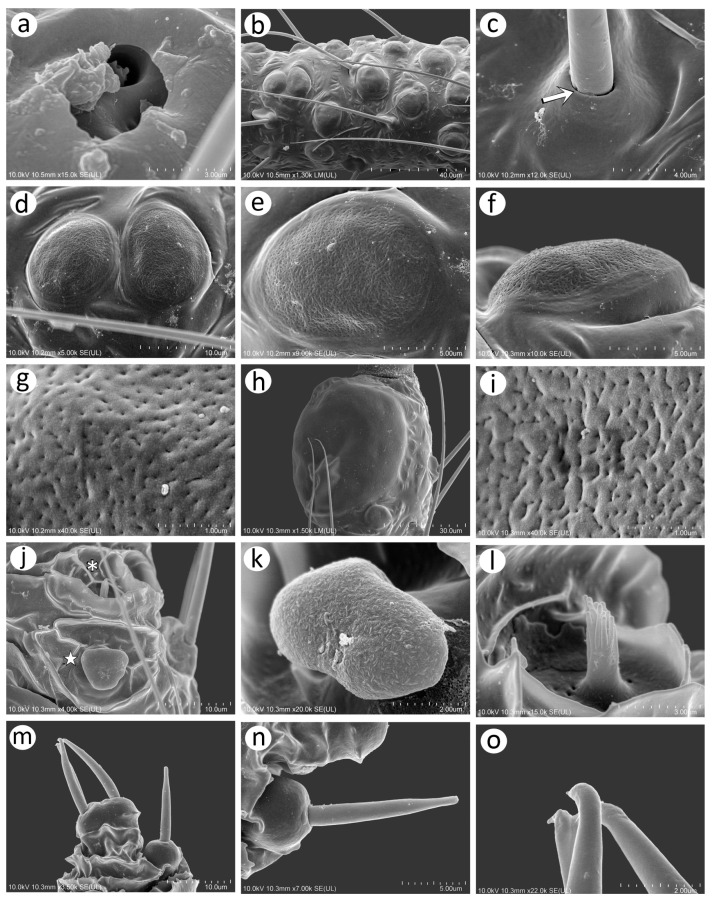
SEM of details of antennal sensilla of the alate male *N. piri*: (**a**) ultrastructure of the two rhinariola type B on the pedicel, (**b**) numerous small placoid sensilla and type I trichoid sensilla on ANT III, (**c**) ultrastructure of the trichoid sensillum socket and basal part with visible moulting pore (arrow), (**d**,**e**) size and shape of the small multiporous placoid sensilla dorsal view surrounded by sclerotic collar, (**f**) lateral side of the small multiporous placoid sensillum, (**g**) ultrastructure of the porous surface of the small multiporous placoid sensillum, (**h**) distal part of ANT V with large multiporous placoid sensillum, (**i**) ultrastructure of the porous surface of the large multiporous placoid sensillum, (**j**) small multiporous placoid sensillum (star) and type II sunken coeloconic sensillum (asterisk) on ANT VI surrounded by sclerotic reinforcements, (**k**) ultrastructure of small multiporous placoid sensillum on ANT VI, (**l**) ultrastructure of type II sunken coeloconic sensillum on ANT VI, (**m**) type II trichoid sensilla on the apical part of the terminal process, (**n**) ultrastructure of the type II trichoid sensillum, (**o**) ultrastructure of the apical parts of the type II trichoid sensilla.

The antennae of the male are characterised by numerous long, fine, pointed type I trichoid sensilla on all segments. The pedicel, like that of the oviparous female, bears a campaniform sensillum and (type I and type II) rhinariola (Figure 9a and Figure 10a). Flagellar segments (ANT III, IV, V and VI) bear numerous small, rounded secondary rhinaria (small multiporous placoid sensilla) uniformly distributed over the entire length and surface of each segment (Figure 9b). Antennal segment V besides small multiporous placoid sensilla bears quite a large multiporous placoid sensillum on the distal part (Figure 9c). The last antennal segment bears small multiporous placoid sensilla (secondary rhinaria), primary rhinaria in the form of one large multiporous placoid sensillum (major rhinarium), two small mushroom-shaped placoid multiporous sensilla (accessory rhinaria), and four sunken coeloconic sensilla, two of type I and two of type II (accessory rhinaria). On the apical part of terminal process type II, trichoid sensilla can be found (Figure 9d).

Type I trichoid sensilla are long and tubular, arising from rounded and flexible sockets (Figure 10b,c). Small multiporous placoid sensilla (secondary rhinaria) are protuberant, mostly rounded or oval, 11–12 μm long, and 9–10 μm wide (Figure 10d). Each sensillum is surrounded by a well-developed sclerotic collar and clearly visible porous membrane (Figure 10e,f). Pores of the small multiporous placoid sensilla are numerous, rounded or elongated, and about 15–20 per μm^2^ (Figure 10g). The big multiporous placoid sensillum is enlarged, oval, 43–45 μm long, and 38–42 μm wide. In its porous membrane, many pores are elongated, 25–30 per μm^2^ (Figure 10h,i). Accessory rhinaria (small multiporous placoid sensilla and coeloconic sensilla) on ANT VI are surrounded by clearly visible sclerotic collars (Figure 10j). Small multiporous placoid sensilla on ANT VI are mushroom-shaped, oval, about 5 μm long, and about 3 μm wide (Figure 10k). Type I sunken coeloconic sensilla are exposed, lying in a flat cavity, and are characterised by 12–14 long projections (Figure 10l). Type II trichoid sensilla are stiff, tubular, tapering in the apical part with semi-spherical sockets (Figure 10m,n) and scattered apices (Figure 10o).

**Figure 11:**
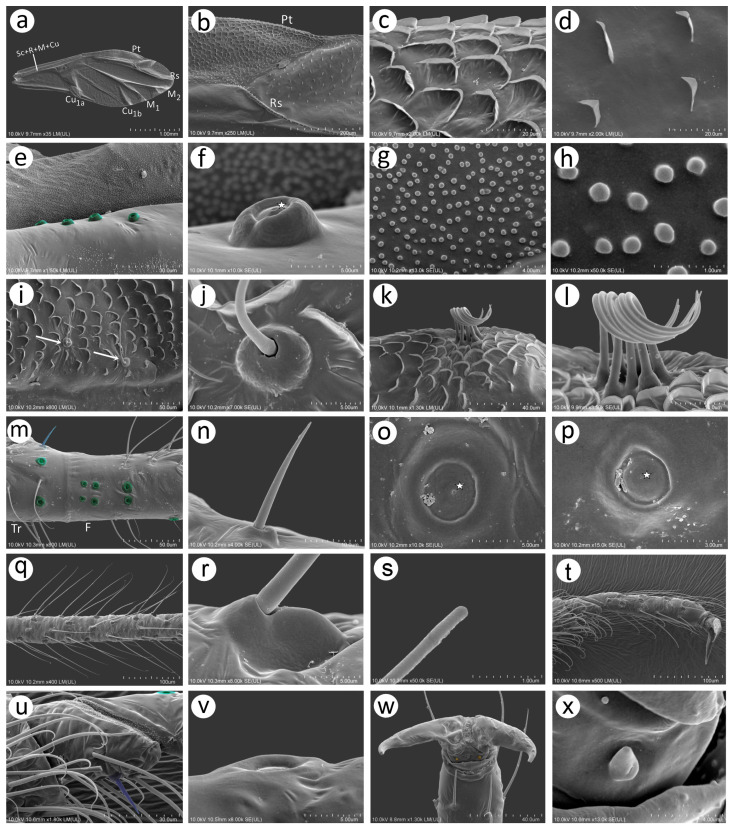
SEM of wings and hind legs of the male *N. piri*: (**a**) general view of the fore wing, (**b**) distal part of pterostigma and proximal part of radial sector, (**c**) numerous crescent-shaped reinforcements on the PT edge, (**d**) scale-like elements on the wing membrane, (**e**) protuberant campaniform sensilla on the wing base, (**f**) ultrastructure of the campaniform sensillum with clearly visible pore (star), (**g**,**h**) numerous minute spherical structures of the wing membrane on the basal part of the wing, (**i**) trichoid sensilla on the lower edge of the pterostigma (arrows), (**j**) ultrastructure of the socket and basal part of the sensillum, (**k**) claval area of the hind wing with numerous reinforcements and 7 hamuli, (**l**) ultrastructure of the claval area hamuli, (**m**) inner side of hind trochanter (TR) and femur (F) showing campaniform sensilla (green) and one short trichoid sensillum on the trochanter ventral side, (**n**) ultrastructure of trichoid sensillum on the trochanter ventral side, (**o**,**p**) ultrastructure of two sizes of campaniform sensilla, (**q**) trichoid sensilla on hind tibia, (**r**) ultrastructure of the trichoid sensilla socket), (**s**) ultrastructure of the apical part of the trichoid sensillum, (**t**) hind tarsus, (**u**) first segment of hind tarsus with one peg-like seta, (**v**) ultrastructure of campaniform sensillum on the dorsal basal part of HT II, (**w**) claws and residual parempodia (yellow), (**x**) ultrastructure of the residual parempodium.

Wings of the male *N. piri* are hyaline, the media are one-branched (Figure 11a), and the pterostigma end is pointed (Figure 11b). The wing membrane is covered with more or less concentrated scale-like elements, which are most numerous on the pterostigma (Figure 11c) and rarer in the other parts of the wing (Figure 11d). The basal part of the wing near the articulation bears evidently protuberant campaniform sensilla, which are more similar to those on the trochanter and femur than the one on the antennal pedicel (Figure 11e). The sensillum is slightly asymmetric and the collar groove is clearly visible (Figure 11f). The wing membrane near the wing articulation is, moreover, densely covered with numerous minute spherical structures (Figure 11g,h). Besides campaniform sensilla on the wing basal part, a few trichoid sensilla have been found along the pterostigma proximal line (Figure 11i). The sensilla are tubular, fine, and with pointed apices and are characterised by hemispherical, rounded, and flexible sockets (Figure 11j). The claval area of the hind wings is characterised by more robust scale-like elements and seven long rolled up hamuli (Figure 11k,l). Campaniform sensilla can also be found on the legs, trochanter, and femora. The hind trochanter besides trichoid sensilla bears, moreover, two campaniform sensilla and one shorter and stiffer sensillum on the ventral side, which may be a chaetic sensillum. Near the trochantro-femoral suture, seven campaniform sensilla may be found—six on the inner side and one on the much outer and dorsal side (Figure 11m). The chaetic-like sensillum is about 20 μm long and arises from a flexible socket (Figure 11n). Larger campaniform sensilla (8–9 μm) are characterised by a more robust collar, while in the smaller ones (4–5 μm), the collar is slimmer. In sensilla of both sizes, the pore lies in the centre of the cap (Figure 11o,p). The legs are densely covered with trichoid sensilla, which are very long, fine, and pointed, and arise from their flexible sockets at an acute angle (Figure 11q,r). In high magnification (about 50K), their apices turn out to be rounded (Figure 11s). Hind tarsi are covered with similar trichoid sensilla; on the first tarsal segment, one peg-like sensillum can be found (Figure 11t,u), and on the dorsal proximal part of the HT II flat, campaniform sensilla are visible (Figure 11v). Parempodia like in the oviparous female are barely visible and residual (Figure 11w,x).

**Figure 12:**
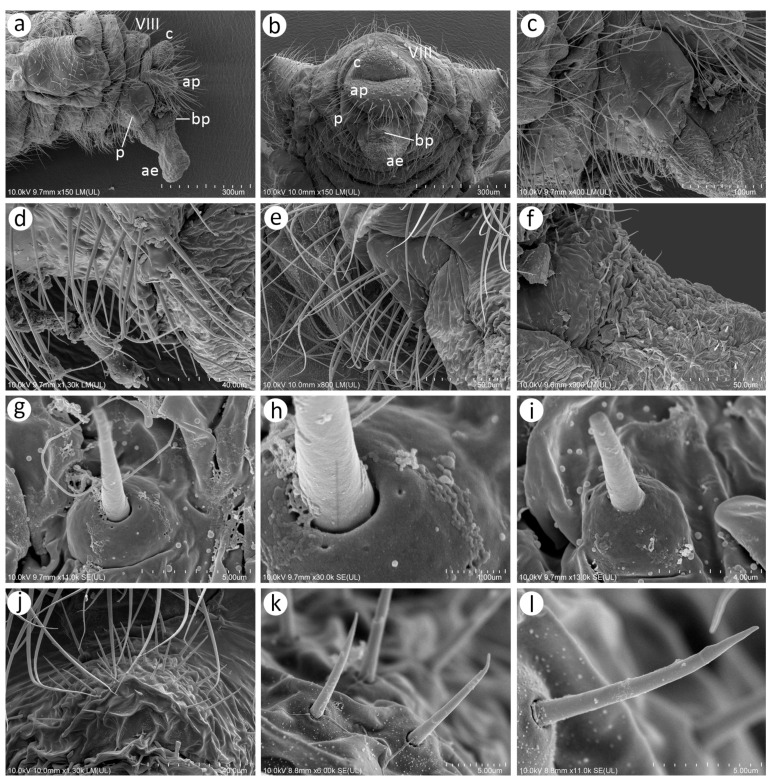
SEM of male genitalia of *N. piri*: (**a**) lateral side of the end of abdomen showing abdominal tergite VIII (VIII), cauda (c), anal plate (ap), parameres (p), basal part of the phallus (bp), and aedeagus (ae), (**b**) rear view of the abdomen showing abdominal tergite VIII (VIII), cauda (c), anal plate (ap), parameres (p), basal part of the phallus (bp), and aedeagus (ae), (**c**) parameres, (**d**) chaetotaxy of parameres in the lateral view, (**e**) chaetotaxy of parameres in the rear view, (**f**) lateral view of the basal part of the phallus and proximal part of aedeagus with numerous short sensilla chaetica, (**g**–**i**) ultrastructure of the sensilla chaetica, (**j**) sensilla chaetica on the basal part of the phallus from the rear view, (**k**,**l**) ultrastructure of sensilla chaetica on the rear of the basal part of the phallus.

The perianal area structures of the male, especially the cauda, anal plate, and genitalia, are extremely setose (Figure 12a). The parameres are robust and sclerotised, lie laterally to the basal part of the phallus and anal plate, and the lower area is more densely covered, with probably trichoid sensilla, than the upper area (Figure 12b,c). The trichoid sensilla on the parameres have similar properties to those on the rest of the body—they are long, tubular, arise from flexible sockets, and have pointed apices (Figure 12d,e). In contrast, the area of the basal part of the phallus and the proximal part of the aedeagus is covered with many short sensilla which may be chaetic sensilla (Figure 12f). They are irregularly distributed on the lateral sides, are shorter, and their apices are more rounded (Figure 12g–i), while from the rear side, they are longer, seem to be stiffer, and are also evidently more pointed (Figure 12j–l). Almost all of them arise from flexible sockets.

### 3.3. Checklist of Species of the Genera Nippolachnus Matsumura, 1917 and Indolachnus gen. nov. Presented in This Work

 Genus *Nippolachnus* Matsumura, 1917
 =*Neonippolachnus* Shinji, 1924 **syn**. **nov**.
**1.**  *Nippolachnus bengalensis* Basu and Hille Ris Lambers, 1968
 =*benzalensis* Basu and Hille Ris Lambers, 1968
 =*benzalensis* Ghosh AK, 1974
**2.**  *Nippolachnus chakrabartii* **sp**. **nov**.
**3.**  *Nippolachnus malayaensis* **sp**. **nov**.
**4.**  *Nippolachnus micromeli* Shinji, 1924
 =*micromelli* Shinji, 1941
 =*Neonippolachnus* betulae Shinji, 1924 **syn**. **nov**.
**5.**  *Nippolachnus piri* Matsumura 1917 **Type species**
 =pyri Takahashi, 1950
**6.**  *Nippolachnus sinensis* **sp**. **nov**.
**7.**  *Nippolachnus xitianmushanus* Zhang and Zhong, 1982 **stat**. **rev**.
 Genus *Indolachnus* **gen**. **nov**.
**1.**  *Indolachnus himalayensis* (van der Goot, 1917) **comb**. **nov**.
 =*Nippolachnus eriobotryae* Basu and Hille Ris Lambers, 1968

**Diagnosis**. Representatives of the genus *Nippolachnus* are easily recognisable from other Tuberolachnini and Lachninae genera. In life, apterous viviparous females are oval, pale green or completely pale without visible sclerotisation, and very densely covered with long, hair-like, fine, pointed, unpigmented setae. *Alate viviparous females* are characterised by characteristic patterns of sclerotisation on the abdomen and membranous parts covered in wax. All morphs feed on the leaves of their host plants, never on woody parts. In mounted specimens, besides the mentioned characters, representatives of this genus are characterised by the absence of an ocular tubercle, but the residual triommatidium is located under the compound eyes. Ultimate rostral segments are wider at the proximal part. The distal part of URS is blunt with blunt, button-shaped RV. Siphuncular sclerites are unpigmented. The first segments of the tarsi have 1-1-1 sense pegs.

**Description.**

*Apterous viviparous females.* Body slender, very delicate, and pale with only yellow or dusky pigmented hind legs. Body densely covered with long, very fine, pointed, unpigmented setae. Head with visible epicranial suture. Antennae six-segmented, sometimes with one small secondary rhinarium on ANT III and IV. Antennal setae always much longer than the width of segments. Primary rhinaria without sclerotised rims. Accessory rhinaria on ANT VI located on the PT (except *N. bengalensis*, where the rhinaria are on the BASE) and also often moved to the lateral side of the segment in relation to the major rhinarium. Terminal process imbricated, poorly separated from BASE without short, rigid subapical setae, and, except *N. bengalensis*, with long fine setae like those on BASE. Head and thorax pale, membranous, without distinct border between the tagmas. Sclerotised part of rostrum groove poorly developed, pale, not more than 0.60 mm. Mesosternal furca delicate, pale, almost separated, fused only by a very thin base without a stem. In some species legs yellow with dusky hind tibiae. Setae on legs long, fine and pointed, longer than the width of tibiae. First segments of tarsi with dorsal length much shorter than the basal length. Abdomen membranous, pale. Siphunculi on low, delicate, and unpigmented and hardly visible sclerites with rather large rounded pores. Genital and anal plates very poorly sclerotised, unpigmented, hardly visible. Cauda rounded.

*Alate viviparous females*. In life, brownish green to dark brown (especially head and thorax) with dusky to brownish legs. In mounted specimens, head and thorax strongly sclerotised, light brown to brown. Body densely covered with long, fine, pointed, unpigmented setae. Head with epicranial suture and large, distinct compound eyes without ocular tubercles, and residual triommatidia located under the compound eyes. Antennae short, with large primary and secondary rhinaria. Secondary rhinaria on ANT III, IV, and V rather rounded, protuberant, almost as wide as the width of segments, in one row on the entire length of the segment. Primary rhinaria oval, larger than the width of segments. Fore wings hyaline. Pterostigma yellow or light brown, short and slightly blunt. Rs bent in the middle of its length, running to the wing apex. Media once-branched except *N. bengalensis*, in which media may be simple or once-branched. Cubital veins straight, arising separately. Legs usually pigmented. Abdomen with characteristic patterns of sclerotisation, in form of differently developed spino-pleural patch on ABD IV–V, pleural patches on ABD II–III, and marginal plates on ABD I–V. Additionally, cross-bars on ABD VI–VII may be present. Siphunculi on clearly visible, pigmented sclerites.

#### 3.3.1. Key to Apterous Viviparous Females of *Nippolachnus* and *Indolachnus* Gen. nov.

**1.**  Body pear-shaped, legs uniformly black, body setae pigmented, PT with one short, thick, rigid subapical seta, LS/BD III 2.40–2.45 … ***Indolachnus himalayensis*** (van der Goot) comb. nov.
**-**  Body slender or narrow-oval, legs never black, setae unpigmented, PT without or with long, fine, pointed setae but never with short, thick, rigid seta, LS/BD III 3.00–7.50 … **2** (*Nippolachnus* Matsumura).
**2.**  Hind legs uniformly yellow without darker parts, PT/BASE 0.27–0.32, BASE with 14–15 setae, URS with 8–10 accessory setae, PT without long, fine setae (only with apical setae) … ***N. bengalensis*** Basu and Hille Ris Lambers.
**-**  Hind legs pale or light brown, often with brown or dark distal parts of femora and tibiae, PT/BASE 0.41–0.69, BASE with 16–27 setae, URS with 14–25 accessory setae, PT with 1–6 long, fine, pointed setae … **3**.
**3.**  Hind femora with dark distal ends, HT I basal length/HT I dorsal length 4.00–4.50, LS/BD III 3.00–3.66, HW/ANT 0.64–0.71 … ***N. chakrabartii*** sp. nov.
**-**  Hind femora without dark distal ends, HT I basal length/HT I dorsal length 1.20–2.66, LS/BD III 4.07–7.50, HW/ANT 0.44–0.64 … **4**.
**4.**  URS with 14–16 accessory setae, ABD VIII with 36–42 setae, hind tibiae yellow or pale without or with light brown distal ends … **5**.
**-**  URS with 18–25 accessory setae, ABD VIII with 45–52 setae, hind tibiae yellow or pale with brown or dark distal ends … **6**.
**5.**  HW/ANT 0.44–0.48, ANT VI/ANT III 0.66–0.81, URS/ANT VI 0.51–0.66, HT II/ANT VI 0.80–1.00 … *N. malayaensis* sp. nov.
**-**  *HW/ANT 0.62–0.63*, *ANT VI/ANT III 0.56–0.63*, *URS/ANT VI 0.81–1.00*, *HT II/ANT VI 0.72–0.75* … ***N. sinensis*** sp. nov.
**6.**  Hind tibiae with light brown to brown distal ends, BASE with 20–22 setae, PT with 4–6 long, fine, pointed setae … ***N. micromeli*** Shinji.
**-**  Hind tibiae with dark brown distal ends, BASE with 25–27 setae, PT with 2–4 long, fine, pointed setae … **7**.
**7.**  ANT with only distal part of ANT VI light brown, HT I with 8–9 setae, ANT VI/ANT III 0.61–0.66 … ***N. xitianmushanus*** Zhang and Zhong.
**-**  ANT with distal parts of ANT IV, V, and VI light brown, HT I with 11–12 setae, ANT VI/ANT III 0.52–0.60 … ***N. piri*** Matsumura.

#### 3.3.2. Key to Alate Viviparous Females of *Nippolachnus* and *Indolachnus* (al. viv. Fem of *N. malayaensis* Unknown)

**1.**  ANT with numerous small secondary rhinaria on the whole area of segments, media of fore wings twice-branched … ***Indolachnus himalayensis*** (van der Goot) comb. nov.
**-**  ANT with not more than 12 large secondary rhinaria (on ANT III) in one row. Media of fore wing unbranched or one-branched … **2** (*Nippolachnus* Matsumura).
**2.**  Media of fore wings unbranched, ABD sclerotisation poorly developed and only in the spinal area … **3**.
**-**  Media of fore wings one-branched, ABD sclerotisation well developed in spinal, pleural, and marginal areas … **4**.
**3.**  ANT III with 8–12 secondary rhinaria of two sizes, ANT IV with 4–5 secondary rhinaria, BASE with 10 setae, HW/ANT 0.54–0.55 … ***N. bengalensis*** Basu and Hille Ris Lambers.
**-**  ANT III with 7–8 secondary rhinaria of one size, ANT IV with 1–2 secondary rhinaria, BASE with 19–21 setae, HW/ANT 0.68–0.69 … ***N. chakrabartii*** sp. nov.
**4.**  ABD VI and VII with spinal and spino-pleural cross-bars … ***N. micromeli*** Shinji.
**-**  ABD VI and VII without sclerites and cross-bars … **5**.
**5.**  ABD with only spinal sclerotisation, ABD IV without marginal sclerites, fore wing with scale-like elements only in the distal part … ***N. sinensis*** sp. nov.
**-**  ABD with only spino-pleural sclerotisation, ABD IV with marginal sclerites, fore wing with scale-like elements also in the middle of the membrane … **6**.
**6.**  Media splitting in front of Rs arising, marginal sclerites on ABD IV not scattered, ABD VIII with 21–24 setae, URS with 30–33 accessory setae … ***N. xitianmushanus*** Zhang and Zhong.
**-**  Media splitting behind Rs arising, marginal sclerites on ABD IV scattered, ABD VIII with 30–32 setae, URS with 18–20 accessory setae … ***N. piri*** Matsumura.

#### 3.3.3. Key to Known Alate Males of *Nippolachnus*:

**1.**  ABD VII with spino-pleural cross-bar, ANT III with 53–64, ANT V with 20–25, ANT VI with 12–16 secondary rhinaria, HT II/ANT VI 0.73–0.86 … ***N. micromeli** *Shinji.
**-**  ABD VII with spino-pleuro-marginal cross-band, ANT III with 95–115, ANT V with 26–35, ANT VI with 21–28 secondary rhinaria, HT II/ANT VI 0.67–0.70 … ***N. piri*** Matsumura.

#### 3.3.4. Key to Known Oviparous Females of Nippolachnus:

**1.**  BL 2.87–3.40, URS with 27–30 accessory setae, PT/BASE 0.54–0.58, HT II/ANT VI 1.21–1.26 … ***N. micromeli*** Shinji.
**-**  BL 4.12–4.25, URS with 15–18 accessory setae, PT/BASE 0.37–0.46, HT II/ANT VI 1.04–1.13 … ***N. piri*** Matsumura.

#### 3.3.5. Key to Known Fundatrices of *Nippolachnus*:

**1.**  Hind femora with light brown patches on distal ends, ANT VI/ANT III 0.48–0.55, HT II/ANT III 0.54–0.57 … ***N. micromeli*** Shinji.
**-**  Hind femora without light brown patches on distal ends, ANT VI/ANT III 0.58–0.63, HT II/ANT III 0.66–0.72 … ***N. piri*** Matsumura.

#### 3.3.6. Review of Species of the Genus *Nippolachnus*

***Nippolachnus* bengalensis Basu and Hille Ris Lambers, 1968**

 =*Nippolachnus benzalensis* Basu and Hille Ris Lambers [20], p. 9
 =*N. benzalensis* Ghosh AK, 1974 [29]

(Figure 13, Figure 14, Figure 15, Figure 16, Figure 17, Figure 18, Figure 19 and Figure 20; Table 1 and Table 2)

**Figure 13:**
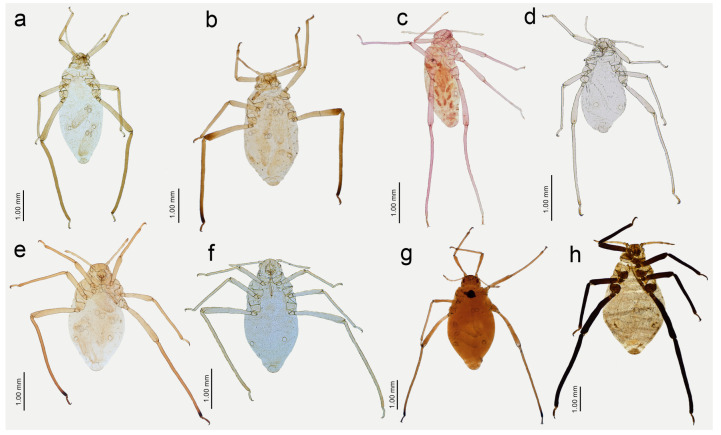
Apterous viviparous females of *Nippolachnus* and *Indolachnus*: (**a**) *N. bengalensis*, (**b**) *N. chakrabartii* sp. nov., (**c***) N. malayaensis* sp. nov., (**d**) *N. micromeli*, (**e**) *N. piri*, (**f**) *N. sinensis* sp. nov., (**g**) *N. xitianmushanus*, (**h**) *I. himalayensis*.

**Figure 14:**
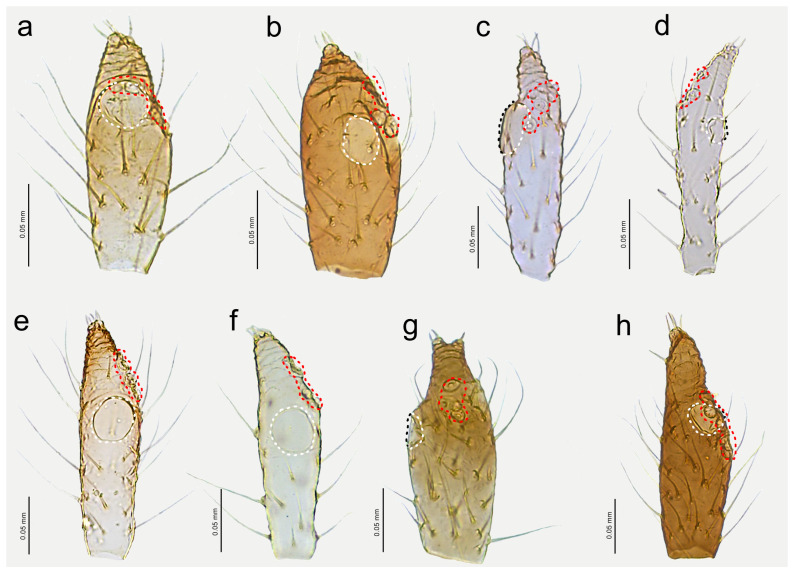
Last antennal segment of apterous viviparous females: (**a**) *N. bengalensis*, (**b**) *N. chakrabartii* sp. nov., (**c**) *N. malayaensis* sp. nov., (**d**) *N. micromeli*, (**e**) *N. piri*, (**f**) *N. sinensis* sp. nov., (**g**) *N. xitianmushanus*, (**h**) *I. himalayensis*; white dotted line indicates the major rhinarium, red dotted line indicates the position of accessory rhinaria relative to the major rhinarium.

**Figure 15:**
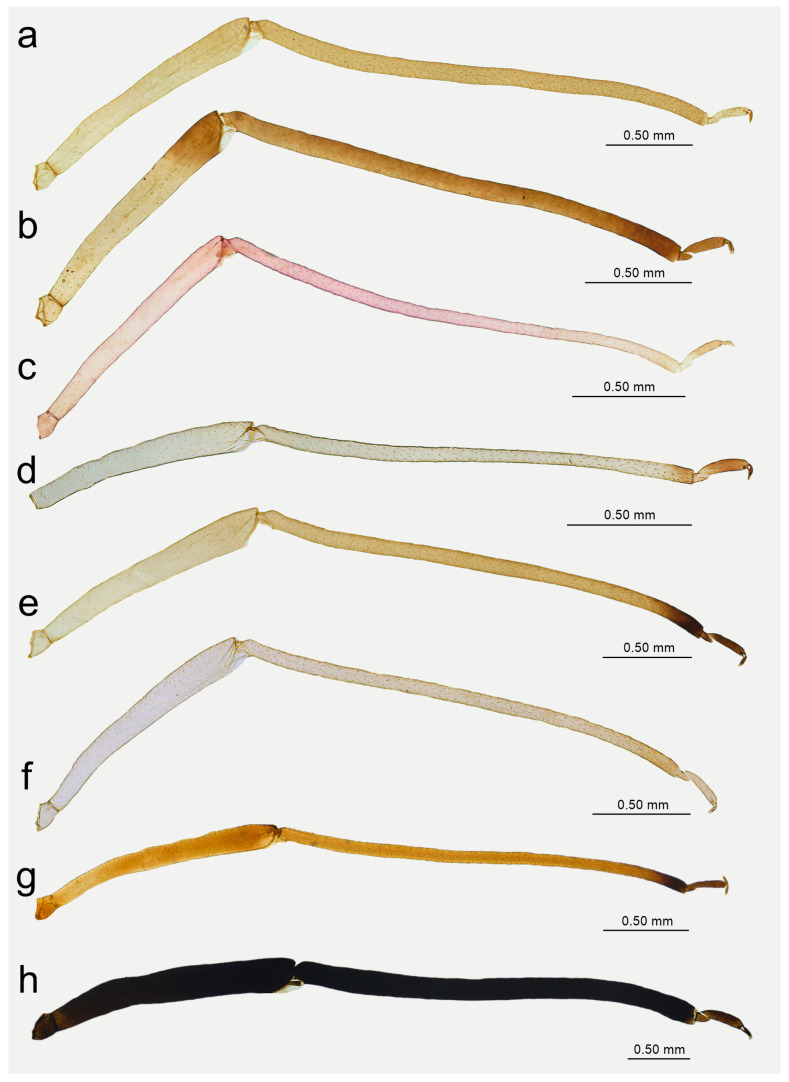
Hind leg pigmentation of apterous viviparous females: (**a**) *N. bengalensis*, (**b**) *N. chakrabartii* sp. nov., (**c**) *N. malayaensis* sp. nov., (**d**) *N. micromeli*, (**e**) *N. piri*, (**f**) *N. sinensis* sp. nov., (**g**) *N. xitianmushanus*, (**h**) *I. himalayensis*.

**Figure 16:**
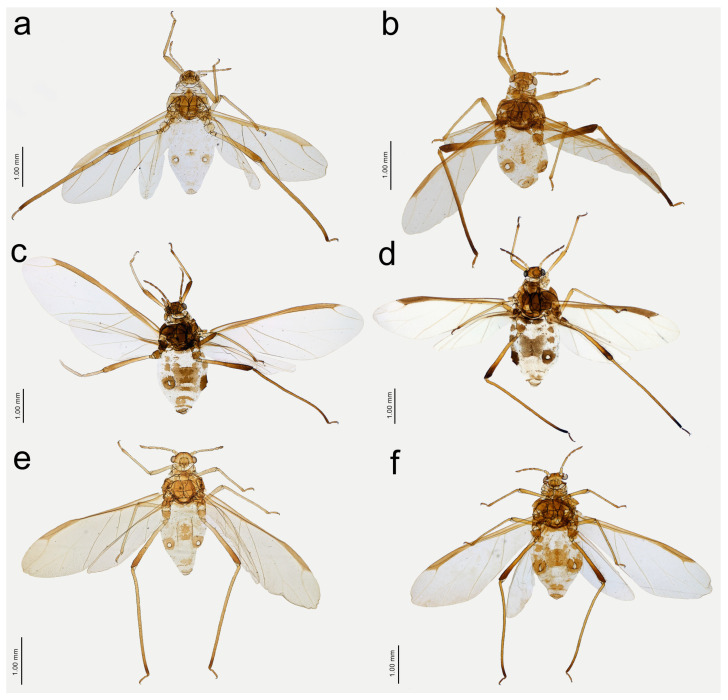
Alate viviparous females of *Nippolachnus*: (**a**) *N. bengalensis*, (**b**) *N. chakrabartii* sp. nov., (**c**) *N. micromeli*, (**d**) *N. piri*, (**e**) *N. sinensis* sp. nov., (**f**) *N. xitianmushanus*.

**Figure 17:**
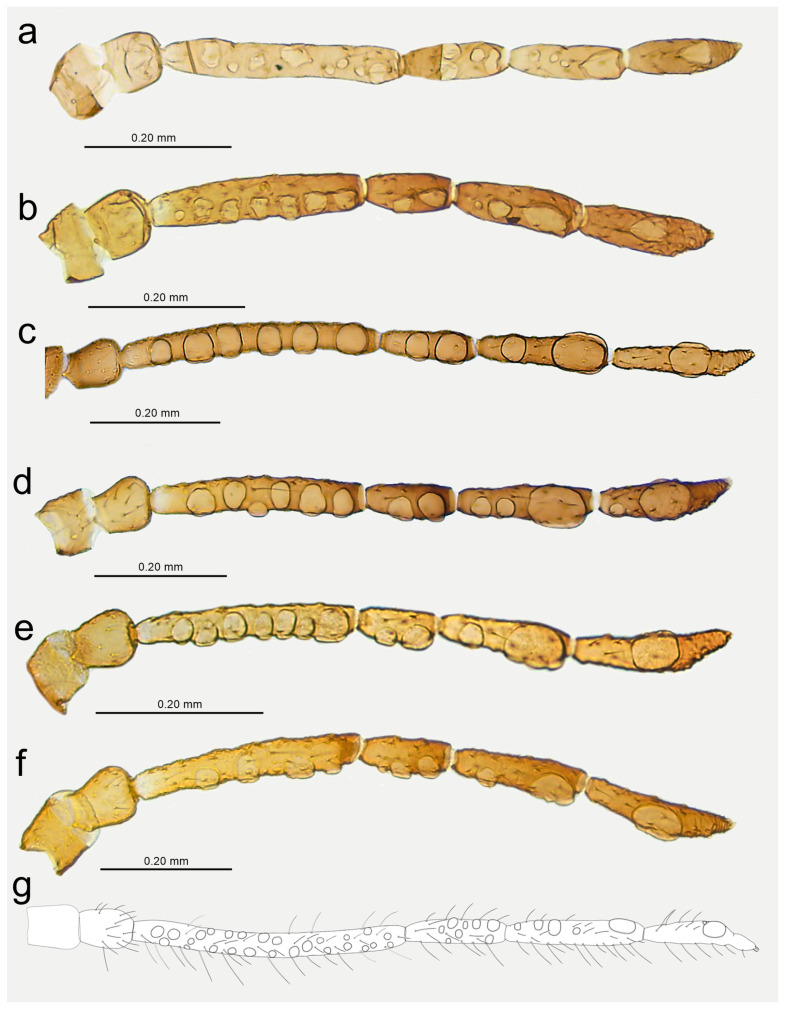
Antennae of alate viviparous females of *Nippolachnus* and *Indolachnus*: (**a**) *N. bengalensis*, (**b**) *N. chakrabartii* sp. nov., (**c**) *N. micromeli*, (**d**) *N. piri*, (**e**) *N. sinensis* sp. nov., (**f**) *N. xitianmushanus*, (**g**) *I. himalayensis* (after van der Goot, redrawn).

**Figure 18:**
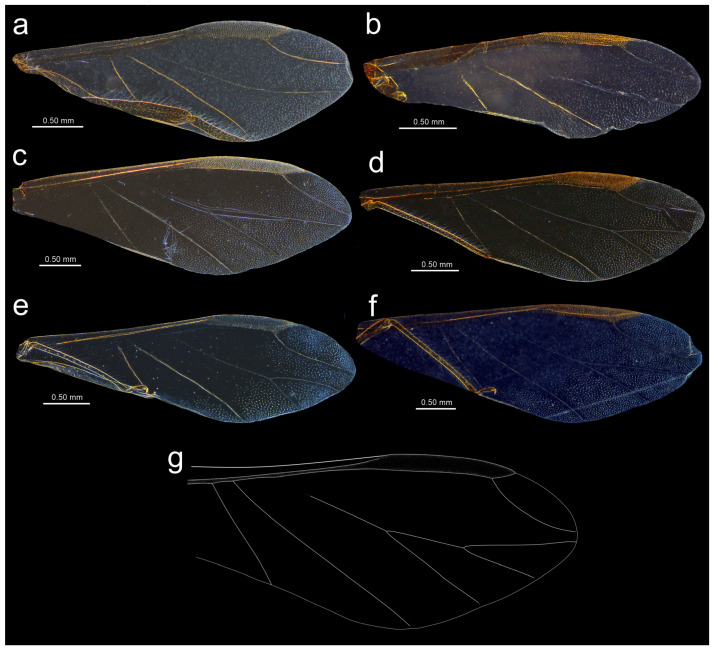
Fore wing venation and scale-like element distribution in alate viviparous females (dark field microscopy): (**a**) *N. bengalensis*, (**b**) *N. chakrabartii* sp. nov., (**c**) *N. micromeli*, (**d**) *N. piri*, (**e**) *N. sinensis* sp. nov., (**f**) *N. xitianmushanus*, (**g**) *I. himalayensis* (after van der Goot, redrawn).

**Table 1:** Measurements (in mm) of apterous viviparous females of *Nippolachnus* and *Indolachnus*.

Character	*Nippolachnus*							*Indolachnus* *himalayensis*
	*bengalensis*	*chakrabartii*	*malayaensis*	*micromeli*	*piri*	*sinensis*	*xitianmushanus*	
BL	2.96–3.62	2.65–3.32	2.45–2.85	2.25–2.27	2.27–3.55	2.35–2.85	3.35	3.45–4.72
HW	0.45–0.50	0.50–0.60	0.44–0.45	0.44–0.50	0.50–0.59	0.50–0.54	0.55	0.65–0.85
ANT	0.91–1.03	0.77–0.89	0.92–1.00	0.75–0.84	0.90–1.03	0.79–0.86	0.85–0.87	1.44–1.51
ANT III	0.33–0.40	0.25–0.27	0.30–0.33	0.24–0.29	0.30–0.34	0.27–0.29	0.28–0.30	0.53–0.58
ANT IV	0.13–0.15	0.10–0.12	0.11	0.07–0.10	0.10–0.14	0.08–0.10	0.11	0.20–0.22
ANT V	0.16–0.18	0.15–0.16	0.16–0.18	0.13–0.15	0.16–0.19	0.15	0.12–0.15	0.25–0.27
ANT VI	0.14–0.16	0.14–0.17	0.21–0.26	0.14–0.18	0.17–0.20	0.15–0.18	0.185	0.22–0.23
BASE	0.11–0.12	0.10–0.11	0.13–0.18	0.09–0.12	0.11–0.13	0.10–0.12	0.11–0.12	0.15
PT	0.03–0.04	0.04–0.06	0.08–0.09	0.04–0.06	0.05–0.07	0.05–0.06	0.06–0.07	0.07–0.08
URS	0.14–0.15	0.16–0.18	0.13–0.14	0.13–0.15	0.17–0.20	0.15	0.18	0.22–0.23
III FEMORA	1.42–1.45	1.12–1.35	1.07–1.17	0.90–1.12	1.20–1.60	0.98–1.27	1.37–1.45	1.08–2.05
III TIBIAE	2.72–2.75	2.15–2.50	2.18–2.47	1.70–2.15	2.37–2.82	2.00–2.37	2.50–2.62	2.87–3.30
HT II	0.22–0.23	0.20–0.22	0.20–0.22	0.19–0.22	0.22–0.25	0.20–0.21	0.22–0.23	0.35–0.36

**Table 2:** Measurements (in mm) of alate viviparous females of *Nippolachnus* and *Indolachnus*. Available measurements of *I. himalayensis* after Basu and Hille Ris Lambers (1968).

Character	*Nippolachnus*						*Indolachnus* *himalayensis*
	*bengalensis*	*chakrabartii*	*micromeli*	*piri*	*sinensis*	*xitianmushanus*	
BL	2.99–3.20	1.91–2.77	2.87–3.25	2.90–4.05	2.62–2.82	3.12–3.95	3.95–4.01
HW	0.50	0.57–0.58	0.56–0.64	0.55–0.70	0.55–0.62	0.66–0.78	-
ANT	0.90–0.96	0.83–0.84	0.98–1.04	0.92–1.16	0.82–0.87	0.95–1.08	1.57
ANT III	0.32–0.35	0.25–0.27	0.32–0.35	0.31–0.39	0.26–0.28	0.30–0.35	0.57–0.67
ANT IV	0.13–0.14	0.10–0.11	0.12–0.14	0.11–0.15	0.09–0.10	0.13–0.16	0.24–0.27
ANT V	0.15–0.17	0.16–0.17	0.18–0.21	0.16–0.19	0.15–0.16	0.19–0.20	0.26–0.27
ANT VI	0.14–0.15	0.16–0.17	0.19–0.20	0.18–0.23	0.18–0.19	0.18–0.23	0.24
BASE	0.11–0.12	0.10–0.11	0.13	0.12–0.16	0.12–0.13	0.12–0.15	0.17
PT	0.03–0.04	0.055	0.06–0.07	0.06–0.07	0.06	0.06–0.08	0.07
URS	0.17–0.19	0.15–0.16	0.15–0.17	0.17–0.19	0.15–0.17	0.19–0.21	0.17–0.19
III FEMORA	1.35–0.37	1.20	1.37–1.60	1.37–1.75	1.17–1.32	1.40–1.70	-
III TIBIAE	2.65–2.72	2.35	2.50–2.82	2.60–3.30	2.30–2.50	2.50–3.05	-
HT II	0.22–0.23	0.20–0.21	0.15–0.17	0.21–0.25	0.21–0.22	0.23–0.24	-

**Figure 19:**
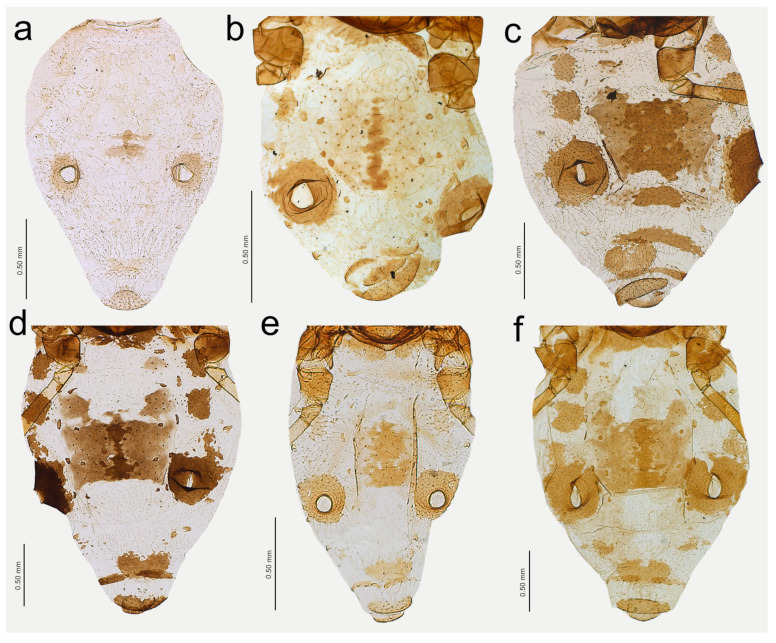
Abdomen sclerotisation of alate viviparous females of *Nippolachnus*: (**a**) *N. bengalensis*, (**b**) *N. chakrabartii* sp. nov., (**c**) *N. micromeli*, (**d**) *N. piri*, (**e**) *N. sinensis* sp. nov., (**f**) *N. xitianmushanus*.

**Figure 20:**
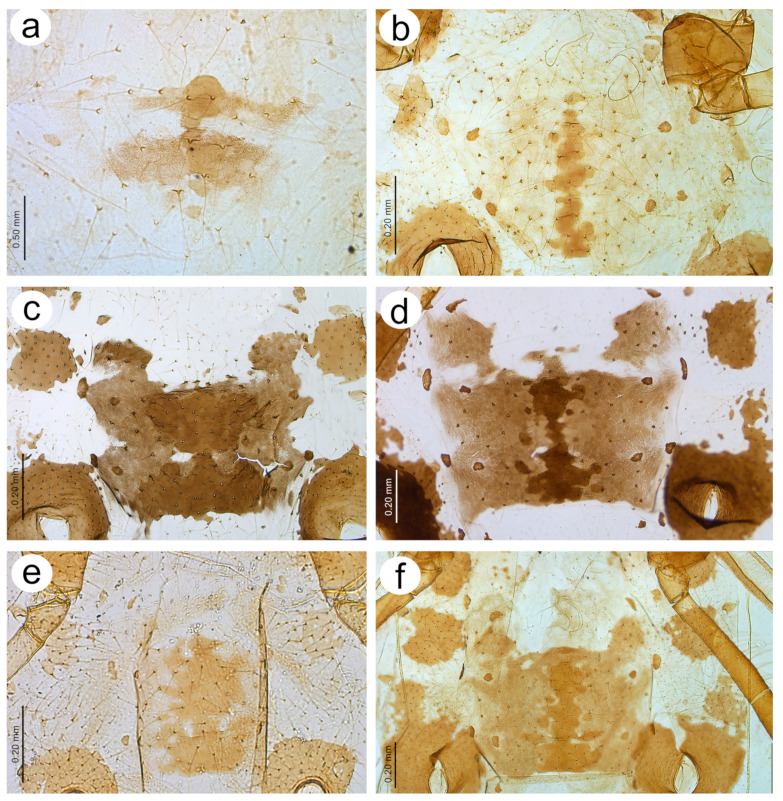
Characters of the central abdominal sclerotisation of alate viviparous females of *Nippolachnus*: (**a**) *N. bengalensis*, (**b**) *N. chakrabartii* sp. nov., (**c**) *N. micromeli*, (**d**) *N. piri*, (**e**) *N. sinensis* sp. nov., (**f**) *N. xitianmushanus*.

**Apterous viviparous** female—re-description

*Colour*: in life, light green [20]. Mounted specimens: body in general pale. ANT pale with yellowish ANT VI. Fore and middle legs pale yellow. Uniformly yellow (slightly darker than fore and middle legs) with paler tarsi (Figure 13a and Figure 15a). *Morphometric characters*: HW 0.48 × ANT. ANT 0.27–0.32 × BL. ANT III with 0–2 (rarely) secondary rhinaria, ANT IV with 0–3 (rarely). ANT V longer than ANT VI with 0–2 (rarely) secondary rhinaria. ANT VI with 14–15 basal setae and without long, fine setae on PT (Figure 14a). ANT VI PT 0.27–0.32 × BASE. Other antennal ratios: VI:III 0.37–0.42, V:III 0.45–0.48, IV:III 0.35–0.41. LS III 5.00–6.00 × BD III. URS 0.37–0.40 × ANT III, 0.90–0.96 × ANT VI, and 0.64–0.65 × HT II with 8–10 accessory setae. Hind tibiae setae 0.08–0.18 mm long. HT I with 7–9 ventral setae (except sense peg). HT II 0.57–0.62 × ANT III and 1.39–1.50 ANT VI. Dorsal setae, 0.11–0.13 mm long. ABD VIII with 40–42 setae.

**Alate viviparous** female—re-description

*Colour*: in life, head and thorax greenish brown, abdomen pale green [20]. Mounted specimens: antennae yellow with darker apical part of ANT V and ANT VI, fore and middle legs uniformly yellow, hind legs yellow with slightly darker apical half of femora, and brown very distal ends of tibiae, tarsi yellow. ABD with light brown SIPH sclerites and small spinal sclerotisation on ABD IV and V (Figure 16a). *Morphometric characters*: HW 0.54–0.55 × ANT. ANT 0.28–0.32 × BL. ANT III with 8–12, ANT IV shorter than ANT V with 4–5 secondary rhinaria. ANT V as long as or longer than ANT VI with 1–5 rounded secondary rhinaria (Figure 17a). ANT VI with 10–12 basal setae and without long, fine setae on PT. ANT VI with PT 0.25–0.36 × BASE, 0–1 small secondary rhinarium. Other antennal ratios: VI:III 0.40–0.46, V:III 0.46–0.51, IV:III 0.37–0.43. LS III about 5.20 × BD III. URS 0.51–0.59 × ANT III, 1.13–1.35 × ANT VI, and 0.75–0.82 × HT II with 13 accessory setae. Fore wings with unbranched media. Scale-like elements distributed on the wing membrane in the distal and cubital part of the wing (Figure 18a). Hind tibiae setae 0.13–0.21 mm long. HT I with 12 ventral setae. HT II 0.61–0.77 × ANT III and 1.06–1.33 ANT VI. Dorsal setae 0.11–0.14 mm long. Abdominal sclerotisation: ABD I with hardly visible spinal sclerites, ABD II-IV without marginal sclerites, only with more setose areas. ABD IV-V with small poorly sclerotised spinal area. Less sclerotised part of the patch on ABD IV and V cuticle wrinkled irregularly (Figure 19a and Figure 20a). ABD VIII with 27–34 setae.

**Material examined**. Paratypes. INDIA, West Bengal, Darjeeling, 26 May 1958, *Eriobotrya dubia*, two apt. viv. fem., one al. viv. fem., five nymphs, S. Das leg., BM 1984-340 (Basu 200) NHMUK; 06 Jan1958, two apt. viv. fem., one nymph, BM 1984-340 (Basu 181) NHMUK; other material: INDIA, West Bengal, Darjeeling 15 Nov. 1968, *E. dubia*, S.G. Rajasingh leg., BM 1984-340 NHMUK.

**Diagnosis**. This species is similar in the pigmentation of the legs of apterous viviparous females to *N. piri* and *N. chakrabartii* sp. nov., but may be easily distinguished from all other species in the genus *Nippolachnus* by the lack of long, fine setae on the PT. *Alate viviparous females* may be distinguished from other morphs by antennae with smaller secondary rhinaria and hardly developed sclerotisation on abdomen.

**Host plants**. ***Eriobotrya dubia***, **Photinia arguta**. Mandal et al. [30] also reported this species from *Quercus* sp. It is most likely that the records on *Pyrus communis*, *P. pashia* should refer to *N. chakrabartii* sp. nov.

**Distribution**. India: Himachal Pradesh, Meghalaya, West Bengal. Holman [9] also mentioned a record from China, after Mandal et al. [30], although in the latter paper there is no record about *N. bengalensis* in China.

***Nippolachnus chakrabartii*** sp. nov.

(Figure 13, Figure 14, Figure 15, Figure 16, Figure 17, Figure 18, Figure 19 and Figure 20; Table 1 and Table 2)

**Apterous viviparous** female—description

*Colour*: in life, unknown. Mounted specimens: body in general yellow. Antennae with light brown ANT IV, V, and VI. Fore and middle legs with light brown distal part of tibiae and tarsi. III FEMORA pale in the proximal part, then yellow and with distinct brown pigmentation at the distal ends (more on the dorsal side). III TIBIAE brown with distinct dark brown distal ends and brown tarsi (Figure 13b and Figure 15b). *Morphometric characters*: HW 0.64–0.71 × ANT. ANT 0.26–0.29 × BL. ANT III with 0–1 secondary rhinarium, ANT IV shorter than ANT V with 0–1 secondary rhinarium. ANT V as long as or minimally shorter than ANT VI. ANT VI with 16–18 basal setae and 1–2 long, fine setae on PT (Figure 14b). ANT VI PT 0.45–0.54 × BASE. Other antennal ratios: VI:III 0.55–0.62, V:III 0.57–0.60, IV:III 0.37–0.46. LS III 3.00–3.66 × BD III. URS 0.61–0.68 × ANT III, 1.08–1.10 × ANT VI, and 0.72–0.82 × HT II with 18 accessory setae. Hind tibiae setae 0.07–0.18 mm long. HT I with 10–11 ventral setae (except sense peg). HT II 0.75–0.84 × ANT III and 1.32–1.51 ANT VI. Dorsal setae, 0.11–0.16 mm long. ABD VIII with 40–44 setae.

**Alate viviparous female**—description

*Colour*: in life, unknown. Mounted specimens: antennae uniformly brown with only slightly lighter basal part of ANT III. Fore and middle legs uniformly yellow to light brown with slightly darker distal parts of femora, tibiae, and tarsi. III FEMORA brown with paler basal part and dark brown distal half. III TIBIAE brown with dark brown distal 1/3 of their length (Figure 15b). *Morphometric characters*: HW 0.68–0.79 × ANT. ANT 0.29–0.43 × BL. ANT III with 7–8, ANT IV shorter than ANT V with two secondary rhinaria. ANT V as long as ANT VI with two secondary rhinaria (Figure 17b). ANT VI with 19–21 basal setae and 2–3 long, fine setae on PT. ANT VI with PT 0.47–0.52 × BASE. Other antennal ratios: VI:III 0.59–0.68, V:III 0.59–0.68, IV:III 0.40. LS III 3.66 × BD III. URS 0.59–0.60 × ANT III, 0.88–1.00 × ANT VI and 0.71–0.80 × HT II with 20–22 accessory setae. Fore wings with unbranched media. Scale-like elements distributed on the wing membrane in the distal part of Cu1a area and in the distal half of the M area of the wing (Figure 18b). Hind tibiae setae 0.08–0.18 mm long. HT I with 12 ventral setae. HT II 0.74–0.84 × ANT III and 1.23–1.25 ANT VI. Dorsal setae 0.11–0.14 mm long. Abdominal sclerotisation: ABD I with spino-pleural cross-band and small marginal sclerites, ABD II and III only with marginal sclerites, ABD IV-V only with small, broken spinal sclerites. ABD VII with small scattered marginal sclerites or scleroites at setal bases, ABD VIII with broken cross-band. Less sclerotised part of the patch on ABD IV and V with pale and hardly visible cuticle (Figure 19b and Figure 20b). ABD VIII with 28–33 setae.

**Material examined**. Holotype. INDIA, West Bengal, Kalimpong, 02 Oct. 1958, *Pyrus* sp., one apt. viv. fem. marked with a circle and “H” letter, S. Das leg., BM 1984-340 (3) NHMUK; Paratypes. One al. viv. fem., other data the same as in holotype, BM 1984-340 (3) NHMUK; one apt. viv. fem., one al. viv. fem., other data the same as in holotype, BM 1984-340 (1) NHMUK; one apt. viv. fem., other data the same as in holotype, BM 1984-340 (4) NHMUK; 07 Mar. 1958, one apt. viv. fem., two nymphs, other data as in holotype, BM 1984-340 (2) NHMUK; one ap. viv. fem., one nymph, BM 1984-340 (5) NHMUK; two apt. viv. fem., BM 1984-340 (6) DZUS;

**Diagnosis**. In the pigmentation of hind tibiae, the new species is similar to *N. bengalensis* and *N. piri*. Apterous viviparous females of the new species can be easily distinguished from both species first of all by the pigmentation of the legs: (**1**) fore and middle tibiae with darker apical parts (fore and middle tibiae uniformly pigmented in *N. bengalensis* and *N. piri*), (**2**) hind femora with brown distal part (hind femora uniformly pigmented). From *N. bengalensis*, apterae may be additionally distinguished by (**1**) 1–2 long setae on the PT (PT of *N. bengalensis* without long setae), (**2**) URS with 18 accessory setae (8–10 accessory setae in *N. bengalensis*), (**3**) PT base 0.45–0.54 (0.27–0.32 in *N. bengalensis*), (**4**) URS/ANT III 0.61–0.68 (0.37–0.40 in *N. bengalensis*). Additionally, apterae of *N. chakrabartii* sp. nov. differ from those of *N. piri* in having the following: (**1**) a smaller number of PT long setae—1–2 (3–4 in *N. piri*); (**2**) ANT V/ANT III 0.57–0.60 (0.47–0.56 in *N. piri*); (**3**) LS/BD III 3.00–3.66 (4.07–7.50 in *N. piri*); (**4**) HT I basal length/H I dorsal length 4.00–4.50 (1.60–2.00 in *N. piri*). *Alate viviparous females* of the new species together with the alatae of *N. bengalensis* differ from those of the other species in having the following: unbranched media of fore wings (once-branched in other *Nippolachnus* and twice-branched in *Indolachnus*); secondary rhinaria as wide as or narrower than half the segment width (secondary rhinaria much wider than half the width of the segment); and poorly developed spinal abdominal sclerotisation. The alate viviparous females of the new species differ from those of *N. bengalensis* in having the following: (**1**) much darker and pigmentation broader pigmentation of III FEMORAE (only slightly darker very distal ends in *N. bengalensis*), (**2**) dark brown 1/3 distal end of III TIBIAE (slightly darker very distal ends in *N. bengalensis*), (**3**) spinal abdominal sclerotisation on ABD III-V (spinal abdominal sclerotisation only on ABD III-IV in *N. bengalensis*), (4) HW/ANT 0.68–0.69 (0.54–0.55 in *N. bengalensis*), (**5**) ANT VI/ANT III 0.59–0.69 (0.40–0.46 in *N. bengalensis*).

**Etymology**. We have the pleasure of naming the new species to honour the outstanding Indian aphidologist Professor Samiran Chakrabarti, our colleague and friend, and for many years the Head of the Department of Zoology, University of Kalyani, India.

**Host plants**. The species has been collected from an unidentified species of *Pyrus* tree.

**Distribution**. Representatives of the new species are so far known from Kalimpong in West Bengal (India).

***Nippolachnus malayaensis sp. nov.***
 *Nippolachnus piri* Takahashi, 1950 [31], p. 592

(Figure 12, Figure 13 and Figure 14; Table 1)

**Apterous viviparous female**—description

*Colour*: in life, unknown. Mounted specimens: body pale with pale appendages. Only ANT VI PT, distal end of hind tibiae and hind tarsi light brown (Figure 13c and Figure 15c). *Morphometric characters*: HW 0.44–0.48 × ANT. ANT 0.34–0.40 × BL. ANT IV always shorter than ANT V. ANT V always shorter than ANT VI. ANT VI with 17–21 basal setae and 1–2 long, fine setae on PT (Figure 14c). ANT VI PT 0.44–0.69 × BASE. Other antennal ratios: VI:III 0.66–0.81, V:III 0.53–0.54, IV:III 0.33–0.36. LS III 5.50–5.75 × BD III. URS 0.39–0.46 × ANT III, 0.51–0.66 × ANT VI and 0.59–0.70 × HT II with 14–15 accessory setae. Hind tibiae setae 0.05–0.15 mm long. HT I with 8–10 ventral setae (except sense peg). HT II 0.65–0.66 × ANT III and 0.80–1.00 ANT VI. Dorsal setae, 0.09–0.11 mm long. ABD VIII with 36–40 setae.

**Material examined**: Holotype. MALAYSIA, Cameron Highlands, 6 Oct. 1944, *Pyrus granulosa*, one apt. viv. fem. Marked with “H” and circle, R. Takahashi leg., 774/55, BM.1955.799 NHMUK; Paratypes. Two apt. vivi. fem., other data as in holotype, 774/55, BM.1955.799 NHMUK; three apt. vivi. fem. other data as in holotype, 773/55, BM.1955.799 NHMUK.

**Diagnosis**. Apterous viviparous females of *Nippolachnus* malayaensis sp. nov. are most similar to *N. sinensis* sp. nov. in having a very pale body and appendages in which both species differ from *N. bengalensis* and *N. chakrabartii* sp. nov. Both species differ, moreover, in the chaetotaxy of ANT BASE (16–17), URS (15–16), and ABD VIII (36–42) from *N. micromeli*, *N. piri*, and *N. xitianmushanus*, in which the values of those characters are 20–27, 18–25, and 45–52, respectively. *Nippolachnus* malayaensis sp. nov. can be easily distinguished from *N. sinensis* sp. nov. by the following differences: (**1**) HW/ANT 0.44–0.48 (0.62–0.63 in *N. sinensis* sp. nov.), (**2**) ANT VI/ANT III 0.66–0.81 (0.57–0.63 in *N. sinensis* **sp**. **nov**.), (**3**) URS/ANT III 0.39–0.46 (0.51–0.57), (**4**) URS/ANT VI 0.59–0.66 (0.81–1.00 in *N. sinensis* sp. nov.), (**5**) HT II/ANT III 0.65–0.66 (0.72–0.75 in *N. sinensis*), (**6**) HT II/ANT VI 0.80–1.00 (1.13–1.31 in *N. sinensis* sp. nov.), (**7**) HT I basal length/HT I dorsal length 1.20–1.50 (2.05–2.33 in *N. sinensis* sp. nov.).

**Etymology**. The name of the new species is derived from “Malaya”, the former name of Malaysia.

**Host plants**. The species has been collected from *Sorbus granulosa* (=*Pyrus granulosa*).

**Distribution**. So far, the species is known from the Cameron Highlands at 1524 m. above sea level (5000 ft) in Malaysia.

***Nippolachnus micromeli* Shinji, 1924**
 Shinji, 1924: 343
 =N. micromelli Shinji, 1941 [32], p. 227; Shiraki, 1952 [33], p. 103
 =Neonippolachnus betulae Shinji, 1924 syn. nov.

(Figure 2, Figure 4, Figure 13, Figure 14, Figure 15, Figure 16, Figure 17, Figure 18, Figure 19, Figure 20, Figure 21, Figure 22 and Figure 23; Table 1, Table 2 and Table 3)

**Figure 21:**
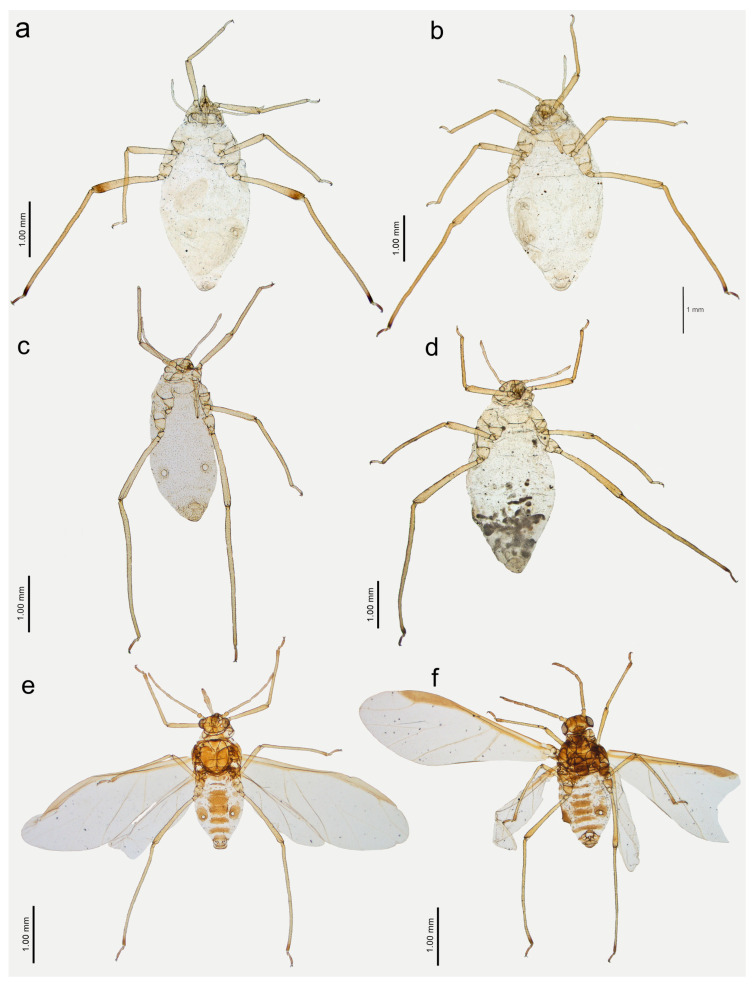
Previously unknown morphs of *Nippolachnus micromeli* and *N. piri*: (**a**) fundatrix of *N. micromeli*, (**b**) fundatrix of *N. piri*, (**c**) oviparous female *N. micromeli*, (**d**) oviparous female *N. piri*, (**e**) alate male *N. micromeli*, (**f**) alate male *N. piri*.

**Table 3:** Measurements (in mm) of fundatrices and sexual morphs of *N. micromeli* and *N. piri*.

Character	*N. micromeli*			*N. piri*		
	Fundatrix	Oviparous Female	Alate male	Fundatrix	Oviparous Female	Alate Male
BL	3.30–4.10	2.87–3.40	2.12–2.50	4.00–4.25	4.12–4.25	2.32–2.90
HW	0.52–0.56	0.48–50	0.50–0.55	0.57–0.65	0.55–0.58	0.56–0.52
ANT	0.86–0.92	0.97–1.01	1.13–1.31	1.05–1.12	1.11–1.16	1.22–1.41
ANT III	0.29–0.33	0.35–0.38	0.44–0.49	0.38–0.42	0.38–0.44	0.47–0.55
ANT IV	0.10–0.11	0.10–0.11	0.13–0.18	0.12–0.13	0.13–0.15	0.16–0.19
ANT V	0.13–0.14	0.18–0.19	0.19–0.26	0.16–0.17	0.18–0.20	0.19–0.23
ANT VI	0.17–0.20	0.18–0.19	0.21–0.23	0.19–0.21	0.22–0.23	0.25–0.29
BASE	0.11–0.13	0.12	0.15–0.16	0.13–0.15	0.15–0.16	0.17–0.20
PT	0.60–0.70	0.60–0.70	0.06–0.07	0.06–0.07	0.06–0.07	0.08–0.09
URS	0.20–0.21	0.18–0.19	0.15–0.17	0.21–0.22	0.20–0.21	0.18–0.20
III FEMORA	1.12–1.22	1.25–1.30	0.90–1.12	1.35–1.50	1.65–1.67	0.95–1.12
III TIBIAE	2.10–2.30	2.42–2.45	1.75–2.00	2.40–2.75	2.90–3.00	1.85–2.17
HT II	0.20–0.20	0.23–0.24	0.17–0.20	0.22–0.23	0.24–0.25	0.17–0.19

**Figure 22:**
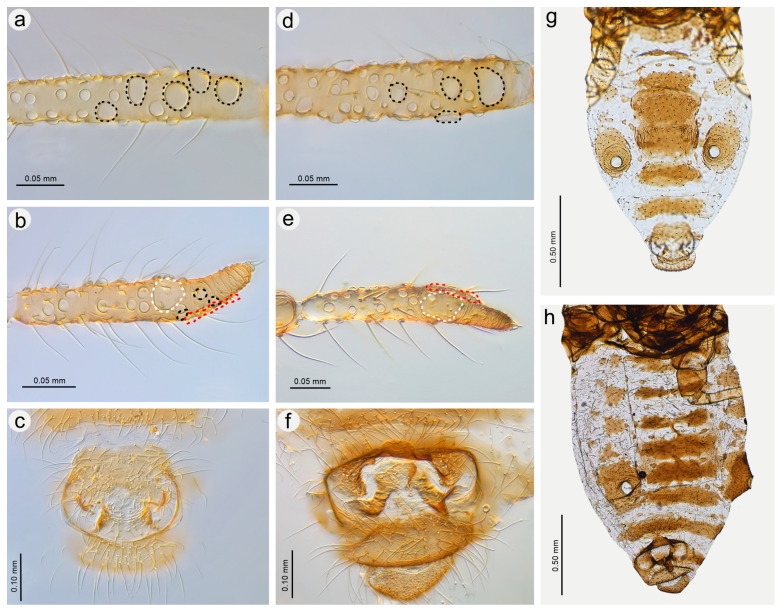
Morphological characters of males: (**a**) antennal sensilla—secondary rhinaria on ANT III of *N. micromeli*, (**b**) antennal sensilla—primary and secondary rhinaria on ANT VI of *N. micromeli*, (**c**) genitalia of *N. micromeli*, (**d**) antennal sensilla—secondary rhinaria on ANT III of *N. piri*, (**e**) antennal sensilla—primary and secondary rhinaria on ANT VI of *N. piri*, (**f**) genitalia of *N. piri*, (**g**) abdomen of *N. micromeli*, (**h**) abdomen of *N. piri*; white dotted line indicates the major rhinarium, red dotted line indicates accessory rhinaria, black line indicates secondary rhinaria on PT.

**Figure 23:**
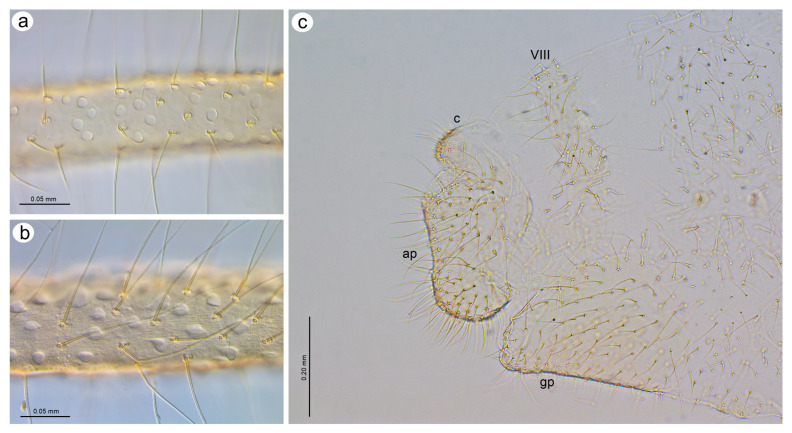
Morphological characters of oviparous females: (**a**) scent plaques of *N. micromeli*, (**b**) scent plaques of *N. piri*, (**c**) perianal structures of oviparous female *N. micromeli*, VIII—abdominal segment VIII, c—cauda, ap—anal plate, gp—genital plate.

**Fundatrix**—description

*Colour*: in life, body in general green. Head light green. Thorax and abdomen green with darker marginal areas and spinal area on abdomen. Antennae and legs pale to light green (Figure 2a,b). Mounted specimens: body pale, ANT pale yellow, fore and middle legs pale yellow, hind femora yellow with brown patches on the distal ends, hind tibiae yellow with dark distal ends and dark tarsi (Figure 21a). *Morphometric characters*: HW 0.56–0.65 × ANT. ANT 0.20–0.28 × BL. ANT IV always shorter than ANT V. ANT V always shorter than ANT VI. ANT VI with 21–24 basal setae and 2–5 long, fine setae on PT. ANT VI PT 0.48–0.59 × BASE. Other antennal ratios: VI:III 0.58–0.65, V:III 0.42–0.46, IV:III 0.31–0.36. LS III 6.00–6.50 × BD III. URS 0.63–0.68 × ANT III, 1.05–1.14 × ANT VI and 0.95–1.00 × HT II with 25–27 accessory setae. Hind tibiae setae 0.08–0.14 mm long. HT I with 10 ventral setae (except sense peg). HT II 0.66–0.72 × ANT III and 1.11–1.14 ANT VI. Dorsal setae, 0.09–0.15 mm long. ABD VIII with 35–46 setae.

**Remarks**: The fundatrix can be easily distinguished from the apterous viviparous females by the following differences: (**1**) larger body 3.30–4.10 mm long (2.25–2.97 in apt. viv. fem.), (**2**) ANT V/ANT III 0.42–0.48 (0.50–0.58 in apt. viv. fem.), (**3**) ANT IV/ANT V 0.75–0.78 (0.50–0.66 in apt. viv. fem.), (**4**) HT II/ANT VI 1.10–1.14 (1.22–1.57 in apt. viv. fem).

**Apterous viviparous female**—re-description

*Colour*: in life, body whitish-green to pale green with pale green appendages and some darker spots in the spinal area of the abdomen, which is the same as in larvae and alatoid nymphs (Figure 2c). Mounted specimens: body pale, almost colourless, only the legs are very pale yellowish with the same pale antennae. III TIBIAE pale yellowish with distal part light brown. HT I pale, HT II brown with paler proximal base (Figure 13d and Figure 15d). *Morphometric characters*: HW 0.55–0.64 × ANT. ANT 0.26–0.37 × BL. ANT III without secondary rhinaria, ANT IV always shorter than ANT V. ANT V shorter or slightly longer than ANT VI. ANT VI with 22–24 basal setae and 4–6 long, fine setae on PT (Figure 14d). ANT VI PT 0.45–0.60 × BASE. Other antennal ratios: VI:III 0.51–0.66, V:III 0.50–0.58, IV:III 0.25–0.34. LS III 4.33–6.00 × BD III. URS 0.48–0.60 × ANT III, 0.80–1.07 × ANT VI and 0.63–0.78 × HT II with 20–25 accessory setae. Hind tibiae setae 0.07–0.16 mm long. HT I with 10–12 ventral setae (except sense peg). HT II 0.70–0.84 × ANT III and 1.22–1.57 ANT VI. Dorsal setae, 0.11–0.15 mm long. ABD VIII with 40–45 setae.

**Alate viviparous female**—re-description

*Colour*: in life, head and thorax dark brown to black with thin wax layer. Antennae dark, legs with brown femora and pale brown tibiae with darker distal ends. Abdomen with dark sclerotisation and white wax powder (Figure 2d). Mounted specimens: body pale, almost colourless, only the legs are very pale yellowish with the same pale antennae. III TIBIAE pale yellowish with distal part light brown. HT I pale, HT II brown with paler proximal base (Figure 16c). *Morphometric characters*: HW 0.56–0.61 × ANT. ANT 0.31–0.34 × BL. ANT III with 5–7 secondary rhinaria, ANT IV shorter than ANT V with 2 secondary rhinaria. ANT V as long as or slightly shorter than ANT VI with 1–3 secondary rhinaria (Figure 17c). ANT VI with 23–26 basal setae and 6–8 long, fine setae on PT. ANT VI with PT 0.50–0.51 × BASE. Other antennal ratios: VI:III 0.51–0.64, V:III 0.50–0.62, IV:III 0.34–0.40. LS III 4.75–6.00 × BD III. URS 0.43–0.46 × ANT III, 0.73–0.85 × ANT VI, and 0.65–0.73 × HT II with 28–30 accessory setae. Media of fore wings once-forked in the middle of the length. Scale-like elements distributed on the wing membrane at distal end of Cu1a, M, and M2 (M1 area uniformly covered) (Figure 18c). Hind tibiae setae 0.08–0.17 mm long. HT I with 12 ventral setae. HT II 0.60–0.71 × ANT III and 1.07–1.23 ANT VI. Dorsal setae 0.11–0.14 mm long. Abdominal sclerotisation: ABD II and III marginal sclerite. On ABD III-V, large spino-pleural patch which is more sclerotised and darker in the spinal area, ABD VI with spinal sclerite and pleural scattered sclerites originating from scleroites at setal bases. ABD VII with spino-pleural cross-band with irregular edges. ABD VIII with spino-pleural cross-band with smooth edges. Less sclerotised part of the patch on ABD III–V cuticle only slightly wrinkled without regular polygons (Figure 19c and Figure 20c). ABD VIII with 38–42 setae.

**Oviparous female**—description

**Figure 24:**
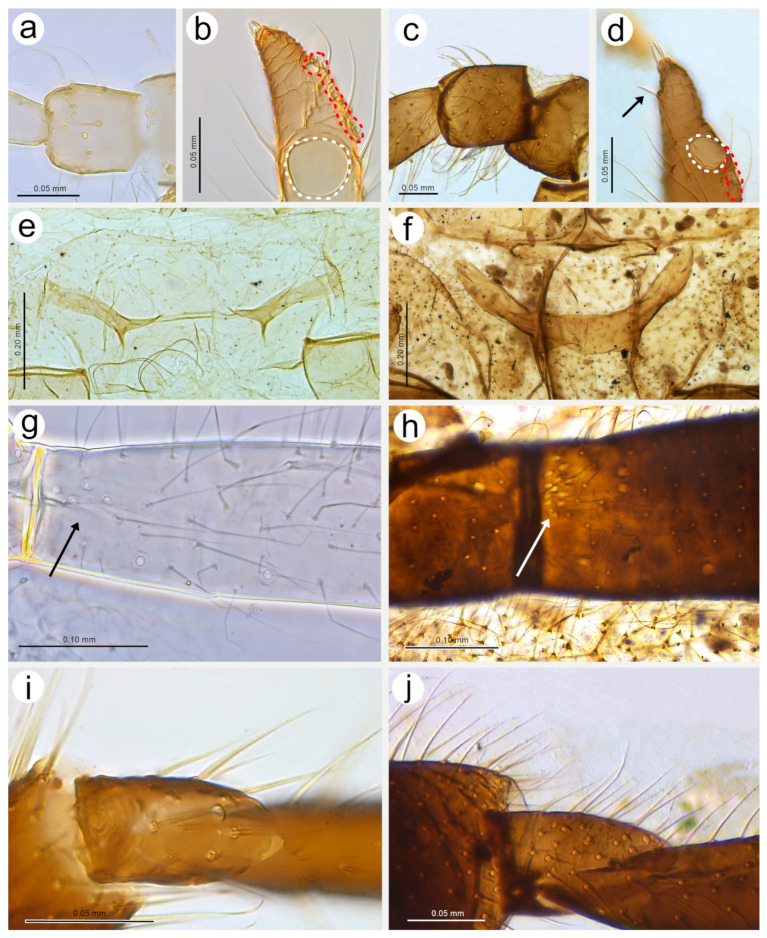
Main morphological differences between genera *Nippolachnus* (**a**,**b**,**e**,**g**,**i**) and *Indolachnus* (**c**,**d**,**f**,**h**,**j**): (**a**) pedicel with few trichoid sensilla, (**b**) terminal process with only long, fine setae (the same as basal setae), at least one small placoid sensillum and all sunken coeloconic sensilla moved from the base, absence of short seta below the apical setae, (**c**) pedicel with numerous trichoid sensilla, (**d**) terminal process only with one short seta below the apical setae and at most one sensillum moved from the base, (**e**) mesosternal furca separated, without stem, delicate, (**f**) mesosternal furca robust, fused with rectangular stem, (**g**) only few campaniform sensilla on hind femur, (**h**) numerous campaniform sensilla on hind femur, (**i**) HT I with few setae, (**j**) HT I with numerous setae; white dotted line indicates the major rhinarium, red dotted line indicates the position of accessory rhinaria relative to the major rhinarium.

*Colour*: in life, body light green with darker patches in the marginal areas of thorax, ABD II, III, and spinal area on ABD IV–V. Legs pale green with pale brown to brown distal ends of tibiae and brown tarsi (Figure 2f,i). Mounted specimens: body pale with yellow appendages (ANT and legs), only hind legs with slightly darker apical ends of tibiae and light brown tarsi (Figure 21c). *Morphometric characters*: HW 0.47–0.51 × ANT. ANT 0.29–0.34 × BL. ANT IV always shorter than ANT V. ANT V shorter or longer than ANT VI. ANT VI with 19–21 basal setae and 6–7 long, fine setae on PT. ANT VI PT 0.54–0.58 × BASE. Other antennal ratios: VI:III 0.48–0.54, V:III 0.48–0.51, IV:III 0.27–0.28. LS III 5.50–6.25 × BD III. URS 0.47–0.51 × ANT III, 0.94–1.00 × ANT VI and 0.75–0.82 × HT II with 27–30 accessory setae. Hind tibiae setae 0.05–0.17 mm long. Hind tibiae poorly swollen with several dozen small, rounded scent plaques (Figure 24a). HT I with 10–12 ventral setae (except sense peg). HT II 0.60–0.68 × ANT III and 1.21–1.26 ANT VI. Dorsal setae, 0.11–0.15 mm long. ABD VIII with 50–60 setae (Figure 24c).

**Alate male**—description

*Colour*: in life, head brown, slightly covered in wax, eyes black, antennae brown. Thorax brown, in some places slightly wax powdered. Legs pale with brown distal ends of hind femora, very distal ends of tibiae and tarsi. Wings hyaline with brown pterostigma. Abdomen greenish with brown to dark sclerotisation (Figure 4g,h). Mounted specimens: head and thorax sclerotised, light brown, ANT yellow with slightly darker apical part of ANT VI, legs yellow with light brown distal ends of tibiae and light brown tarsi, wings hyaline with yellow pterostigma, ABD sclerotisation light brown (Figure 21e). *Morphometric characters*: HW 0.41–0.44 × ANT. ANT 0.46–0.53 × BL. ANT III with 53–64 in general small and rounded secondary rhinaria of which 2–5 on the basal part are much larger than the others (Figure 22a). ANT IV shorter than ANT V with 20–25 secondary rhinaria. ANT V always shorter or longer than ANT VI with 17–22 secondary rhinaria. ANT VI with 12–16 secondary rhinaria, of which few (2–3) are located not only on the BASE but also on PT (Figure 22b), 24–26 basal setae, and 5–8 long, fine setae on PT. ANT VI PT 0.38–0.43 × BASE. Other antennal ratios: VI:III 0.46–0.52, V:III 0.43–0.53, IV:III 0.29–0.36. LS III 3.33–4.00 × BD III. URS 0.34–0.38 × ANT III, 0.65–0.79 × ANT VI and 0.87–1.00 × HT II with 32–34 accessory setae. Hind tibiae setae 0.06–0.13 mm long. HT I with 13–15 ventral setae (except sense peg). HT II 0.38–0.40 × ANT III and 0.73–0.86 ANT VI. Dorsal setae, 0.08–0.11 mm long. ABD VIII with 24–26 setae. Abdomen with spinal sclerites on ABD I–VI, spino-pleural cross-bar on ABD VII and cross-band on ABD VIII. Marginal sclerites on ABD II–IV (Figure 22g). Genitalia sclerotised with robust and setose parameres (Figure 22c).

**Material examined**: Neotype. JAPAN, Amakubo, Tsukuba, 15 Oct. 2016, *Rhaphiolepis indica* var. *umbellata*, one al. viv. fem., M. Miyazaki leg., Jap/16/10/2 DZUS (designated by Kanturski et al. 2018); other material: one al. viv. fem., other data as in Neotype, Jap/16/10/03 DZUS; one al. viv. fem., other data as in Neotype, Jap/16/10/04 DZUS; one ♀, other data as in Neotype, Jap/16/10/01 DZUS; Sapporo, 19 Sep. 1962, *Ulmus japonica* (=*Ulmus davidiana* var. *japonica*), four al. viv. fem., two nymphs, M. Sorin leg., 14,494 MNHN; 21 Sep. 1962, three al. viv. fem., three nymphs, 14,497 MNHN; five al. viv. fem., one nymph, 210,102,830 NIAES, 22 Sep. 1966, *Sorbus alnifolia*, one al. viv. fem., M. Miyazaki leg., 1996 (1) NIAES, one al. viv. fem., 1996 (2) NIAES; 12 apt. viv. fem., M. Sorin leg., 14,502 MNHN; Senami, Murakami, 17 Jan. 1992, *Rhaphiolepis indica* var. *umbellata*, four ♀, K. Baba leg., 14,513 MNHN; two ♀, 210102831, NIAES; Nikko, 28 Sep. 1975, S. alnifolia, five al. viv. fem., M. Sorin leg., 14,506 MNHN; 24 Aug. 1980, one apt. viv. fem., two nymphs, R. Blackman leg., 2141 NHMUK; KOREA, Mt. Oseo, 15 Oct. 2011, *Sorbus alnifolia*, one al. viv. fem., Y. Lee leg., Kor/11/10/01 DZUS; one al. viv. fem., Kor/11/10/02 DZUS; Jeonnan Univ. Arborettum, 7 May 2016, *Rhaphiolepis indica* var. *umbellata*, one al. viv. fem., Y. Lee leg., Kor/16/05/01 DZUS; one al. viv. fem., Kor/16/05/02 DZUS; two al. viv. fem., Kor/16/05/03 DZUS; *R. indica*, two al. viv. fem., Kor/16/05/4; Geumoch Island, 5 Jun. 2016, Sorbus sp., two apt. viv. fem., J. Choi leg., Kor/16/06/1 DZUS; two apt. viv. fem., Kor/16/06/2; Cheollipo 1-gil, Sawon-myeon, Taean-gun, Chungcheongnam-do, 7 Nov. 2020, *Rhaphiolepis indica*, var. *umbellata*, four ♀, three ♂, M. Lee leg., 201107-LMH-1(1–7) SNU; 14 May 2023, three Fx, 230514-LMH-1 (1–3), DZUS.

**Diagnosis**. In pigmentation of mounted specimens, very pale apterous viviparous females of *N. micromeli* are similar to *N. malayaensis* sp. nov. and *N. sinensis* sp. nov. Both new species are characterised by almost pigmentless appendages (especially legs), in which they differ from *N. micromeli* (and other species in which the legs are yellow or with brown distal ends). Additionally, *N. micromeli* differs from the new species in the chaetotaxy of ANT VI BASE (20–22 setae), URS (20–25), and ABD VIII (45–50), which in *N. malayaensis* sp. nov. and *N. sinensis* sp. nov. have 16–21, 14–16, and 36–42 setae, respectively. The large number of setae of remaining body parts makes *N. micromeli* similar to *N. piri* and *N. xitianmushanus* together with darker pigmented distal ends of tibiae. *N. micromeli* differs from both species in the following: (**1**) unpigmented ANT VI (pigmented in *N. piri* and *N. xitianmushanus*); (**2**) 20–22 basal setae (25–27 in *N. piri* and *N. xitianmushanus*); (**3**) 4–6 long setae on PT (3–4 in *N. piri* and *N. xitianmushanus*). Apterous viviparous females of *N. micromeli* differ from those of *N. piri* additionally by having a higher ratio of HT I basal to HT I dorsal length, 2.00–3.50 (1.60–2.00 in *N. piri*), and from those of *N. xitianmushanus* by ANT IV/ANT V 0.50–0.66 (0.73–0.91 in the latter). *Alate viviparous females* can be easily distinguished by abdominal sclerotisation with spinal and spino-pleural cross-bars on ABD VI and VII, which are absent in the known alatae of the other species.

**Host plants**. *Rhaphiolepis indica*, *R. umbellata*, *Sorbus alnifolia*, *Sorbus* sp*. Ulmus davidiana* var*. japonica*.

**Biology**. In the Korean Peninsula, the first representatives of this species—fundatrices and first instar fundatrigeniae—were observed in mid-May on *R. indica* var. *umbellata* (Figure 4a) and with some time, each observed colony exhibited the presence of the fundatrix and alatoid nymphs without apterous female larvae. From the beginning of the colonies’ activity, the aphids were attended by *Crematogaster matsumurai* ants (Figure 4e). In the autumn, the colonies consisted of alate viviparous females, oviparous females, and male nymphs. In late October, alate viviparous females gave birth to the sexual generation, and after mating, the oviparous females laid eggs in a circular and disordered pattern around the main vein on the undersides of the host plant where they overwinter.

**Distribution**. This species is known from Japan and the Korean Peninsula.

**Remarks**. For a long time, *Nippolachnus micromeli* has been treated as a synonym of *N. piri*. Kanturski et al. [13] performed detailed morphological analyses supported by molecular studies and restored *N. micromeli* as a separate species. All previous records of *N. piri* from the above-mentioned host plants should be referred to *N. micromeli*.

***Nippolachnus piri Matsumura*, 1917**
 Matsumura, 1917 [15], p. 382
 (Figure 2, Figure 3, Figure 5, Figure 6, Figure 7, Figure 8, Figure 9, Figure 10, Figure 11, Figure 12, Figure 13, Figure 14, Figure 15, Figure 16, Figure 17, Figure 18, Figure 19, Figure 20, Figure 21, Figure 22 and Figure 23; Table 1, Table 2 and Table 3)

**Fundatrix**—description

*Colour*: in life, head light brown. ANT pale yellowish. Fore legs yellow, middle legs with green-yellowish femora, and pale yellowish tibiae. Hind legs with light green proximal parts, and yellow distal parts of femora and yellow tibiae with dark distal ends and dark tarsi. Dorsal side of body yellow with green marginal areas of thorax and ABD I–VI. On ABD VII and VII, green pleuro-marginal cross-bands. Spinal areas of thorax and abdomen with brown patches, especially on ABD I and ABD IV–VI (Figure 3a,b). Mounted specimens: head slightly sclerotised, yellow, ANT pale yellow with light brown distal part of ANT VI. Fore and middle legs yellow. Hind legs yellow with dark distal ends of tibiae and dark tarsi (Figure 21b). *Morphometric characters*: HW 0.54–0.59 × ANT. ANT 0.25–0.26 × BL. ANT IV always shorter than ANT V. ANT V always shorter than ANT VI. ANT VI with 22–25 basal setae and 0–4 long, fine setae on PT. ANT VI PT 0.40–0.50 × BASE. Other antennal ratios: VI:III 0.48–0.55, V:III 0.40–0.42, IV:III 0.28–0.32. LS III 6.50–8.00 × BD III. URS 0.52–0.57 × ANT III, 1.04–1.07 × ANT VI, and 0.95–1.00 × HT II with 18–22 accessory setae. Hind tibiae setae 0.11–0.17 mm long. HT I with 9–10 ventral setae (except sense peg). HT II 0.54–0.57 × ANT III and 1.04–1.12 ANT VI. Dorsal setae, 0.10–0.16 mm long. ABD VIII with 43–55 setae.

**Remarks**: The fundatrix can be easily distinguished from the apterous viviparous females by the following differences: (**1**) larger body 4.00–4.25 mm long (2.27–3.55 in apt. viv. fem.); (**2**) longer ANT III 0.38–0.42 (0.30–0.34 in apt. viv. fem.); (**3**) ANT V/ANT III 0.40–0.42 (0.47–0.56 in apt. viv. fem.); (4) URS/HT II 0.95–1.00 (0.70–0.86 in apt. viv. fem.).

**Apterous viviparous female**—re-description

*Colour*: in life, head yellowish-light green, ANT pale to light green with light brown PT. Pronotum green, rest of thorax light green with green longitudinal stripes on the margin, fore and middle legs pale green with dusky, hind legs with greenish femora, light brown tibiae and dark tarsi, abdomen light green with green longitudinal marginal stripes, spinal patches on ABD I, IV, and V, and green cross-bars on ABD VII (Figure 2c,d). Mounted specimens: body in general pale. Head pale or yellow, ANT pale with light brown to brown distal part of ANT V and distal part of ANT VI, fore and middle legs pale to yellow, hind femora yellow with pale proximal part, hind tibiae pale yellow to yellow with dark distal end and both segments of dark tarsi (Figure 13e and Figure 15e). *Morphometric characters*: HW 0.54–0.62 × ANT. ANT 0.26–0.42 × BL. ANT III 1.03–1.23 × ANT IV + ANT V with 0–1 secondary rhinarium, ANT IV with 0–1 secondary rhinarium. ANT V shorter or as long as ANT VI. ANT VI with 25–27 basal and 3–4 long, fine setae on PT (Figure 14c). ANT VI PT 0.41–0.54 × BASE. Other antennal ratios: VI:III 0.52–0.60, V:III 0.47–0.56, IV:III 0.30–0.41. LS III 4.07–7.50 × BD III. URS 0.56–0.62 × ANT III, 0.97–1.14 × ANT VI and 0.70–0.86 × HT II with 18–21 accessory setae. Hind tibiae setae 0.06–0.18 mm long. HT I with 8–9 ventral setae (except sense peg). HT II 0.67–0.80 × ANT III and 1.14–1.47 ANT VI. Dorsal abdominal setae, 0.11–0.16 mm long. ABD VIII with 50–55 setae.

**Alate viviparous female**—re-description

*Colour*: in life, head and thorax brown. Antennae dark, femora and tibiae of legs brown with dark distal ends. Abdomen dark brown with white wax patches (Figure 3g). Very freshly moulted specimens with brown head (with darker antennae), yellow thorax, and brown-greenish legs with darker distal ends. Abdomen light green with darker patches in the central and marginal part, without wax (Figure 3e). The freshly moulted specimens after some time become darker and start to be slightly visible on the abdomen (Figure 3f). Mounted specimens: body with brown head, yellowish-brown ANT with brown apex of ANT IV, ANT V, and VI (Figure 1c,d,f). Fore and middle legs uniformly pale yellow to yellow, hind legs with yellow femora, and yellow to light brown tibia with dark distal parts and whole tarsi (Figure 16d). *Morphometric characters*: HW 0.59–0.65 × ANT. ANT 0.28–0.33 × BL. ANT III with 8–11 secondary rhinaria, ANT IV shorter than ANT V with 2–3 secondary rhinaria. ANT V shorter or as long as ANT VI with 1–2 secondary rhinaria (Figure 17d). ANT VI with XX basal setae and 3–4 long, fine setae on PT. ANT VI with PT 0.42–0.50 × BASE. Other antennal ratios: VI:III 0.55–0.60, V:III 0.47–0.55, IV:III 0.35–0.38. LS III 3.20–5.20 × BD III. URS 0.48–0.57 × ANT III, 0.80–1.00 × ANT VI, and 0.70–0.90 × HT II with 18–20 accessory setae. Scale-like elements distributed in the distal part of Cu1a, M1, and M2 areas of the membrane. Media one-branched with the fork in the distal half of the pterostigma length under the Rs rising (Figure 18d). Hind tibiae setae 0.13–0.21 mm long. HT I with 12 ventral setae. HT II 0.61–0.77 × ANT III and 1.06–1.33 ANT VI. Dorsal setae 0.11–0.14 mm long. Abdominal sclerotisation: ABD I with spino-pleural cross-band and small marginal sclerites, ABD II and III with pleural and marginal sclerites and very small sclerites at setal bases between them, ABD IV with marginal sclerites more or less fused with SIPH sclerites and large sclerotic spino-pleural cross-band, ABD V proximal part with spino-pleural cross-band fused with the previous one, ABD VI and VII without sclerites, ABD VIII with pleuro-marginal cross-band. Less sclerotised part of the patch on ABD IV and V cuticle wrinkled or rugose in the form of polygonal reticulation (Figure 19d and Figure 20d). ABD VIII with 30–33 setae.

**Oviparous female**—description

*Colour*: in life, head pale yellow, antennae pale yellow with brown distal part of ANT VI. Prothorax green with yellowish spinal part. The rest of the thorax and abdomen yellowish with green marginal stripes and green spinal patches on metathorax, ABD I, IV, V, VI, and VII. Legs yellowish with brown distal ends of tibiae and tarsi (Figure 3h,i,k). Mounted specimens: head and thorax pale yellow, abdomen pale, ANT yellow with slightly darker apical part of ANT VI, legs yellow with dark apical ends of hind tibiae and dark hind tarsi (Figure 21d). *Morphometric characters*: HW 0.54–0.59 × ANT. ANT 0.25–0.26 × BL. ANT IV always shorter than ANT V. ANT V always shorter than ANT VI. ANT VI with 27–30 basal setae and 3–5 long, fine setae on PT. ANT VI PT 0.37–0.46 × BASE. Other antennal ratios: VI:III 0.50–0.60, V:III 0.45–0.52, IV:III 0.29–0.39. LS III 6.80–10.00 × BD III. URS 0.45–0.52 × ANT III, 0.86–0.95 × ANT VI, and 0.83–0.95 × HT II with 15–18 accessory setae. Hind tibiae setae 0.08–0.20 mm long. Hind tibiae with several dozen small, rounded scent plaques (Figure 23b). HT I with 8–10 ventral setae (except sense peg). HT II 0.54–0.63 × ANT III and 1.04–1.13 ANT VI. Dorsal setae, 0.10–0.16 mm long. ABD VIII with 38–44 setae.

**Alate male**—description

*Colour*: in life, head blackish, slightly covered in wax powder, antennae brown with darker ANT IV-VI. Thorax brown, femora of legs brown with darker distal halves, tibiae pale brown with dark distal ends and dark tarsi. Wing hyaline with brown pterostigma. Abdomen whitish with greenish-brown sclerotised cross-bands (Figure 3j,k). Mounted specimens: head and thorax sclerotised, brown. ANT light brown with slightly darker distal part of ANT V and ANT VI. Legs yellow with brown distal parts of tibiae and brown tarsi of fore and middle legs. Distal ends of hind tibiae and tarsi dark brown. Wings hyaline with brown pterostigma. Abdominal sclerotisation brown (Figure 21f). *Morphometric characters*: HW 0.43–0.45 × ANT. ANT 0.47–0.54 × BL. ANT III with 95–115 small and rounded secondary rhinaria of which 3–4 on the basal part are much larger (Figure 22b), ANT IV always shorter than ANT V with 25–43 secondary rhinaria. ANT V always shorter than ANT VI with 26–35 secondary rhinaria. ANT VI with 21–28 secondary rhinaria also on PT (Figure 22e), 20–24 basal setae, and 3–6 long, fine setae on PT. ANT VI PT 0.40–0.47 × BASE. Other antennal ratios: VI:III 0.50–0.55, V:III 0.40–0.44, IV:III 0.32–0.34. LS III 3.33–4.00 × BD III. URS 0.34–0.38 × ANT III, 0.62–0.72 × ANT VI, and 0.92–1.05 × HT II with 20–25 accessory setae. Hind tibiae setae 0.06–0.15 mm long. HT I with 10 ventral setae (except sense peg). HT II 0.34–0.37 × ANT III and 0.67–0.70 ANT VI. Dorsal setae, 0.10–0.13 mm long. ABD VIII with 17–22 setae. Abdomen with spino-pleural cross-bars on ABD I–VI and cross-bands on ABD VII and VIII. Marginal sclerites on ABD I–IV (Figure 22h). Genitalia strongly sclerotised with both robust and sclerotised parameres and the basal part of phallus (Figure 22f).

**Material examined**: Neotype. JAPAN, Shizuoka-ken, 14 Apr. 2015, *Eriobotrya japonica*, one al. viv. fem., M. Sano leg., Jap/15/04/1 DZUS (designated by Kanturski et al. 2018); Fukoka Univ., 01 May 1964, two al. viv. fem., R. v.d. Bosh leg., BM 1984-340 NHMUK; Tokio, 23 Jul. 1956, *Pyrus* sp., five apt. viv. fem., one al. viv. fem., R. Takahashi leg., BM 1984-340 NHMUK; Mt. Koya, 23 Sep. 1960, *Pyrus* sp., two al. viv. fem., two nymphs, M. Sorin leg., 14,487 MNHN; Kuroyama, Osaka, 21 Jun. 1961, *Pyrus* sp., one apt. viv. fem., one al. viv. fem., M. Sorin leg., 210,102,829 NIAES; Yatabe, 01 Oct.1980, *Pyrus communis*, two apt. viv. fem., M. Miyazaki leg., 3754 (1) NIAES; one al. viv. fem., 3754 (2) NIAES; one al. viv. fem., 3754 (3) NIAES; two al. viv. fem., 3754 (4) NIAES; Fukoka Univ., 21 Apr. 1964, Eriobotrya sp., two al. viv. fem., R. v.d. Bosh leg., BM 1984-340 (1) NHMUK; two al. viv. fem., one nymph, BM 1984-340 (2) NHMUK; two al. viv. fem., one nymph, BM 1984-340 (3) NHMUK; one Fx, one al. viv. fem., BM 1984-340 (4) NHMUK; 15.04.1964, Fukoka Univ., 21 Apr. 1964, two Fx, BM 340-1984 (1) NHMUK; two Fx, BM 340-1984 (2) NHMUK; two Fx, one nymph, BM 340-1984 (3) NHMUK; two fundatrices, BM 340-1984 (4) NHMUK; two Fx, BM 340-1984 (5) NHMUK; KOREA, Chusan experimental forest, 17 Jun. 2016, *Pyrus pyrifolia*, one apt. viv. fem., Y. Lee leg., Kor/16/06/1 DZUS; one apt. vivi. fem., one al. viv. fem., Kor/16/06/2 DZUS; one apt. viv. fem., one al. viv. fem., Kor/16/06/3 DZUS; one apt. viv. fem., one al. viv. fem., Kor/16/06/4 DZUS; Odong-do, 14 Jul. 2014, P. pyrifolia, two apt. viv. fem., Y. Lee leg., Kor/14/07/1; two apt. viv. fem., Kor/14/07/2 DZUS; Sangbal-ri, Yongsan-myeon, Jangheung-gun, Jeollanam-do, 12 Nov. 2020, *Eriobotrya indica*, four ♀, three ♂, M. Lee leg., 221112-LMH-1 (1–7) SNU; Expo-daero, Sora-myeon, Yeosu-si, Jeollanam-do, four Fx, 230513-LMH-1(1–4) SNU.

**Diagnosis**. Apterous viviparous females of *N. piri* are similar to *N. bengalensis*, *N. chakrabartii* sp. nov. and *N. xitianmushanus* in the pigmentation of the legs, in which they differ from the other apterae, which have colourless or only slightly pigmented very distal ends of antennae, tibiae, and tarsi. Apterae of *N. piri* can be easily distinguished from *N. bengalensis* by the much darker distal area of hind tibiae and dark hind tarsi (only slightly darker very distal apices of hind tibiae and yellow tarsi in *N. bengalensis*). From *N. chakrabartii* sp. nov., *N. piri* apterae differ in the lack of dark pigment in the distal part of the hind femora (femora with dark distal patches in *N. chakrabartii* sp. nov.), and ANT V and VI with darker apical halves (ANT IV-VI uniformly brown in *N. chakrabartii* sp. nov.). *Nippolachnus piri* apterae differ from those in *N. xitianmushanus* as follows: (**1**) only ANT VI distal part pigmented (brown pigmented distal part of ANT IV and V and brown ANT VI in *N. xitianmushanus*); (**2**) 8–9 HT I setae (11–12 in *N. xitianmushanus*); (**3**) ANT VI/ANT III 0.52–0.60 (0.61–0.66 in *N. xitianmushanus*). *Alate viviparous females* of *N. piri* are most similar to *N. sinensis* sp. nov. and *N. xitianmushanus*. The three species differ from the other species in abdominal sclerotisation (poorly developed in *N. bengalensis* and *N. chakrabartii* sp. nov. and with sclerotised cross-bars on ABD VI and VII in *N. micromeli*). Alatae of *N. piri* differ from those of *N. sinensis* sp. nov. in having much more developed spino-pleural sclerotisation on ABD III–V, and ABD IV with marginal plates which are fused with SIPH sclerites (ABD III–V mostly with spinal sclerotisation and ABD IV without marginal sclerites in *N. sinensis* sp. nov.). Alatae of *N. piri* differ from those of *N. xitianmushanus* in having the following: (**1**) poorly developed marginal sclerites on ABD VI (well-developed in *N. xitianmushanus*); (**2**) more than 30 setae on ABD VIII (21–25 in *N. xitianmushanus*); (**3**) URS/ANT III 0.48–0.57 (0.58–0.63 in *N. xitianmushanus*).

**Host plants**. Representatives of *N. piri* feed on *Eriobotrya japonica*, *Eriobotrya* sp., *Pyrus communis*, *P*. pyrifolia, *Pyrus* sp.

**Biology**. In mid-May, three colonies of this species were discovered on the undersides of leaves of *Eriobotrya japonica* (Figure 4c). Each colony exhibited one fundatrix, alate viviparous female adults, and nymphs; however, apterous viviparous females were not observed. Two colonies were observed to be approached by *Camponotus japonicus* (Figure 4f), and in one colony, *Pristmyrmex punctatus* (Figure 4h) was observed. Considering the presence of alate viviparous female nymphs in all colonies and the absence of apterous viviparous females, it can be inferred that the second generations likely consist of alate viviparous females for host plant migration. From mid-May, alate viviparous females moved to another host plant species and began forming colonies on *Pyrus pyrifolia* (Figure 4b) in early to mid-June, with apterous viviparous females outnumbering them at a ratio of 9 to 1. On *Pyrus*, the colonies have been attended by *Formica japonica* (Figure 4d,g). At the turn of summer and autumn in September, *Pyrus* colonies of alatoid nymphs and alate viviparous females were observed. Meanwhile, in mid-October, just before returning to Eriobotrya for wintering, the number of late-born females was higher at 9 to 1 compared to June. In November, alate viviparous females that returned to the primary host produced oviparae and males through reproduction. After mating, they laid eggs in a row with consistent spacing on the undersides of leaves near the main vein of the host plant where they overwintered.

**Distribution**. Despite a lot of historic publications in which *N. piri* is described as a part of the fauna (e.g., India), our studies indicate that this species is restricted to Japan, the Korean Peninsula, China (north-eastern part), Taiwan [34], and Laos [35]. This species could be present in the Russian Far East.

**Remarks**. Otake [36] described the oviposition behaviour as well as the survival and hatching of the eggs and fate of young colonies [37,38] of *N. piri* on *Rhaphiolepis umbellta* (=*R. indica*). However, according to Kanturski et al. [13], this plant genus is the host plant of *N. micromeli*, and therefore the results of those publications should refer to this species and not *N. piri*.

***Nippolachnus sinensis* sp. nov.**

(Figure 13, Figure 14, Figure 15, Figure 16, Figure 17, Figure 18, Figure 19 and Figure 20; Table 1 and Table 2)

**Apterous viviparous female**—description

*Colour*: in life, unknown. Mounted specimens: body very pale, only tibiae yellow with pale brown distal ends. Tarsi yellow (Figure 13f and Figure 15f). *Morphometric characters*: HW 0.62–0.63 × ANT. ANT 0.30–0.33 × BL. ANT III with 0–1 secondary rhinarium, ANT IV always shorter than ANT V with 0–1 secondary rhinarium. ANT V as long as or shorter than ANT VI. ANT VI with 16–17 basal setae and 2–3 long, fine setae on PT (Figure 14f). ANT VI PT 0.54–0.55 × BASE. Other antennal ratios: VI:III 0.57–0.63, V:III 0.51–0.55, IV:III 0.31–0.34. LS III 6.25–7.50 × BD III. URS 0.51–0.57 × ANT III, 0.01–1.00 × ANT VI, and 0.71–0.75 × HT II with 15–16 accessory setae. Hind tibiae setae 0.10–0.18 mm long. HT I with about 7 ventral setae (except sense peg). HT II 0.72–0.75 × ANT III and 1.13–1.32 ANT VI. Dorsal setae, 0.13–0.15 mm long. ABD VIII with 38–42 setae.

**Alate viviparous female**—description

*Colour*: in life, unknown. Mounted specimens: head and thorax light brown. Antennae yellow with slightly darker distal end of ANT V and VI. Fore and middle legs yellow with light brown distal ends of femora and tarsi. Hind femora brown with darker distal 1/3. Hind femora yellow to light brown with darker distal ends of tibiae and darker tarsi (Figure 16e). *Morphometric characters*: HW 0.67–0.71 × ANT. ANT 0.30–0.31 × BL. ANT III with 7–8 secondary rhinaria, ANT IV shorter than ANT V with 1–2 secondary rhinaria. ANT V shorter than ANT VI with one secondary rhinarium (Figure 17e). ANT VI with 16–17 basal setae and 2–3 long, fine setae on PT. ANT VI with PT 0.46–0.50 × BASE. Other antennal ratios: VI:III 0.67–0.69, V:III 0.57, IV:III 0.34–0.35. LS III 2.80–3.00 × BD III. URS 0.53–0.65 × ANT III, 0.78–0.94 × ANT VI, and 0.68–0.79 × HT II with about 15–16 accessory setae. Scale-like elements distributed in the very distal part of Cu1a, M1, and M2 areas of the membrane. Media one-branched with the fork in the distal part of the pterostigma length, directly under the Rs rising (Figure 18e). Hind tibiae setae 0.06–0.180 mm long. HT I with 10 ventral setae. HT II 0.78–0.82 × ANT III and 1.15–1.19 ANT VI. Dorsal setae 0.10–0.13 mm long. Abdominal sclerotisation: ABD I with spino-pleural broken cross-band, ABD II with marginal sclerites, ABD III with marginal sclerites and spinal sclerite fused with poorly developed central abdominal sclerotisation, ABD IV with only a few small marginal scleroites at setal bases and large spinal sclerite. ABD VI without sclerites, ABD VII with residual marginal sclerites, ABD III with broken cross-band. Less sclerotised part of the patch on ABD IV and V poorly developed and rather smooth (Figure 19e and Figure 20e). ABD VIII with about 14–17 setae.

**Material examined**. Holotype. CHINA, Junnan, Kunming, 15 May 1980, ***Pyrus pyrifolia***, one apt. viv. fem., Tieseng Zhong and Lin-Yao Wang leg., BM 2004-145 NHMUK; Paratypes. One al. viv. fem., other data as in the holotype, BM 2004-145(2) NHMUK; one al. viv. fem., one nymph, other data as in the holotype, BM 2004-145(1) DZUS; Jilin Province, 29 Sep. 2008, host unknown, one apt. viv. fem., Kim et al. leg., 08/09/01 DZUS.

**Diagnosis**. Apterous viviparous females of *Nippolachnus* sinensis sp. nov. are most similar to *N. malayaensis* sp. nov. in having a very pale body and appendages in which both species differ from *N. bengalensis* and *N. chakrabartii* sp. nov. Both species differ, moreover, in the chaetotaxy of ANT BASE (16–17), URS (15–16), and ABD VIII (36–42) from *N. micromeli*, *N. piri*, and *N. xitianmushanus*, in which the values of those characters are 20–27, 18–25, and 45–52, respectively. *Nippolachnus* sinensis sp. nov. can be easily distinguished from *N. malayaensis* sp. nov. by the following differences: (**1**) HW/ANT 0.62–0.63 (0.44–0.48 in *N. malayaensis* sp. nov.), (**2**) ANT VI/ANT III 0.57–0.63 (0.66–0.81 in *N. malayaensis* sp. nov.), (**3**) URS/ANT III 0.51–0.57 (0.39–0.46 in *N. malayaensis* sp. nov.), (**4**) URS/ANT VI 0.81–1.00 (0.59–0.66 in *N. malayaensis* sp. nov.), (**5**) HT II/ANT III 0.72–0.75 (0.65–0.66 in *N. malayaensis* sp. nov.), (**6**) HT II/ANT VI 1.13–1.31 (0.80–1.00 in *N. malayaensis* sp. nov.), (**7**) HT I basal length/HT I dorsal length 2.05–2.33 (1.20–1.50 in *N. malayaensis* sp. nov.). *Alate viviparous females* differ from other species in having one- branched media (*N. micromeli*, *N. piri* and *N. xitianmushanus*), poorly developed abdominal sclerotisation, e.g., ABD IV without marginal sclerites (present in *N. piri* and *N. xitianmushanus*), and without spino-pleural sclerites on ABD VI and VII (present on *N. micromeli*). The fore wing membrane of *N. sinensis* sp. nov. is, moreover, characterised by less developed scale-like elements distributed in the distal parts of the wing membrane.

**Host plants**. The species has been collected from *Pyrus betulifolia*.

**Distribution**. *Nippolachnus sinensis* sp. nov. is known from two localities in China, in Kunming, and from an unspecified locality in the Jilin Province.

***Nippolachnus xitianmushanus* Zhang and Zhong, 1982 stat. rev.**
 Zhang and Zhong, 1982 [21], p. 25

(Figure 13, Figure 14, Figure 15, Figure 16, Figure 17, Figure 18, Figure 19 and Figure 20; Table 1 and Table 2)

**Apterous viviparous female**—description

*Colour*: in life, unknown. Mounted specimens: body and appendages in general pale and poorly pigmented. ANT yellow with darker distal parts of ANT IV and ANT V. ANT VI light brown with paler proximal part. Fore and middle legs pale to yellow. Hind legs pale to yellow with slightly darker distal ends of femora, dark distal ends of tibiae, and dark tarsi (Figure 13g and Figure 15g). *Morphometric characters*: HW 0.62–0.64 × ANT. ANT 0.25–0.26 × BL. ANT IV shorter than ANT V. ANT V shorter than ANT VI. ANT VI with 25–27 basal setae and 3–4 long, fine setae on PT (Figure 14g). ANT VI PT 0.41–0.54 × BASE. Other antennal ratios: VI:III 0.61–0.66, V:III 0.40–0.53, IV:III 0.36–0.39. LS III 5.80–6.80 × BD III. URS 0.60–0.64 × ANT III, 0.97 × ANT VI, and 0.78–0.81 × HT II with 20 accessory setae. Hind tibiae setae 0.10–0.18 mm long. HT I with 11–12 ventral setae (except sense peg). HT II 0.73–0.82 × ANT III and 1.18–1.24 ANT VI. Dorsal setae, 0.11–0.16 mm long. ABD VIII with 52 setae.

**Alate viviparous female**—re-description

*Colour*: in life, unknown. Mounted specimens: head and thorax brown. Antennae brown with darker distal end of ANT III, darker halves of ANT IV–VI. Fore and middle legs yellow to light brown with slightly darker distal ends of femora and darker tarsi. Hind femora brown with darker distal 1/3. Hind femora yellow to light brown with darker distal ends of tibiae and darker tarsi (Figure 16f). *Morphometric characters*: HW 0.60–0.72 × ANT. ANT 0.27–0.30 × BL. ANT III with 4–10 secondary rhinaria, ANT IV shorter than ANT V with two secondary rhinaria. ANT V shorter than ANT VI with 1–2 secondary rhinaria (Figure 17f). ANT VI with 15–20 basal setae and 4–6 long, fine setae on PT. ANT VI with PT 0.48–0.59 × BASE. Other antennal ratios: VI:III 0.60–0.65, V:III 0.54–0.63, IV:III 0.37–0.47. LS III 4.16–5.60 × BD III. URS 0.58–0.63 × ANT III, 0.91–1.05 × ANT VI, and 0.82–0.87 × HT II with about 33 accessory setae. Scale-like elements distributed in the distal part of Cu1a, M, and M1 areas of the membrane. Media one-branched with the fork in the middle of pterostigma length (Figure 18f). Hind tibiae setae 0.10–0.20 mm long. HT I with 11 ventral setae. HT II 0.68–0.76 × ANT III and 1.04–1.27 ANT VI. Dorsal setae 0.13–0.16 mm long. Abdominal sclerotisation: ABD I with spino-pleural cross-band, ABD II with marginal sclerites, ABD III with marginal sclerites and small pleural sclerite fused with the rest of the central abdominal sclerotisation, ABD IV with marginal sclerites more or less fused with SIPH sclerites and large sclerotic spino-pleural cross-band. Central abdominal sclerotised patch in spinal and pleural position of ABD IV and ABD V. ABD VI without sclerites, ABD VII with marginal sclerites, ABD III with broken cross-band. Less sclerotised part of the patch on ABD IV and V cuticle forms more or less regular polygonal reticulation (Figure 19f and Figure 20f). ABD VIII with about 23–26 setae.

**Material examined**. Lectotype (here designated). CHINA, Zhejiang, Xitianmu Shan, 12 May 1975, *Eriobotrya japonica*, one al. viv. fem. marked with “L”, Tie-sen Zhong and Guang-xue Zhang leg., 5686-1(-1-6), 939,351 IOZ; Paralectotype. One al. viv. fem., other data the same as in Lectotype, 939,357 IOZ; other material. CHINA, Jiangxi, Jiulianshan, 08 Jun. 1975, *Pyrus* sp., one apt. viv. fem., one al. viv. fem., Wang Huifu leg., 6190(-1-3), 939343, 939,337 IOZ; Zhejiang, Xitianmu Shan, 13 May 1975, *Eriobotrya japonica*, two al. viv. fem., Tie-sen Zhong and Guang-xue Zhang leg., 5686-1(-1-1), BM 2004-145(1) NHMUK; one al. viv fem., other data as in the previous number, 5686-1(-1-2), BM 2004-145(2) NHMUK.

**Diagnosis**. Apterous viviparous females of *N. xitianmushanus* are similar to *N. bengalensis*, *N. chakrabartii* sp. nov., *N. micromeli*, and *N. piri* in pigmentation of the legs; they differ from the other apterae in having pale or only slightly pigmented very distal ends of antennae, tibiae, and tarsi. From *N. bengalensis*, apterae of *N. xitianmushanus* can be easily distinguished by the much darker distal area of hind tibiae and dark hind tarsi (only slightly darker very distal apices of hind tibiae and yellow tarsi in *N. bengalensis*). From *N. chakrabartii* sp. nov., *N. xitianmushanus* apterae differ in the lack of dark pigment in the distal part of the hind femora (femora with dark distal patches in *N. chakrabartii* sp. nov.). Apterae of *N. xitianmushanus* differ from *N. micromeli* in having the following: (**1**) pigmented distal part of ANT IV, V, and pigmented ANT VI (only distal end of ANT VI pigmented in *N. micromeli*); (**2**) BASE with 25–27 setae (20–22 in *N. micromeli*); (**3**) HT I basal length/HT I ventral length 0.50–0.53 (0.35–0.46 in *N. micromeli*). The species can be distinguished from *N. piri* by the following: (**1**) pigmented distal part of ANT IV, V, and pigmented ANT VI (only distal part of ANT VI pigmented in *N. piri*); (2) HT I with 11–12 setae (8–9 setae in *N. piri*); (3) ANT VI/ANT III 0.61–0.66 (0.52–0.60 in *N. piri*). *Alate viviparous females* of *N. xitianmushanus* are most similar to *N. piri* and *N. sinensis* sp. nov. The three species differ from the other species in abdominal sclerotisation (poorly developed in *N. bengalensis* and *N. chakrabartii* sp. nov. and with extra sclerotisation on ABD VI and VII in *N. micromeli*). Alatae of *N. xitianmushanus* differ from those of *N. sinensis* sp. nov. by having much more developed spino-pleural sclerotisation on ABD III–V, and ABD IV with marginal plates which are fused with SIPH sclerites (ABD III–V mostly with spinal sclerotisation and ABD IV without marginal sclerites in *N. sinensis* sp. nov.). Alatae of *N. xitianmushanus* differ from *N. piri* in having the following: (1) well-developed marginal sclerites on ABD VI (poorly developed in *N. piri*); (2) 21–25 setae on ABD VIII (more than 30 setae in *N. piri*); (3) URS/ANT III 0.58–0.63 (0.48–0.57 in *N. piri*).

**Host plants**. This species has been described from *Eriobotrya japonica*.

**Distribution**. So far, the species is known from its type locality in Xitianmu Shan, Zhejang in China.

**Remarks**: Probably due to its short description in Chinese, *Nippolachnus xitianmushanus* has been treated as a possible synonym of *N. piri* by Blackman and Eastop [10,11]. This status was also upheld by Remaudière and Remaudière [19]. Comparative analyses of the type material of this and other species and the listed differences clearly show the independent status of this species, which was also corroborated in the results of the phylogenetic analyses.

#### 3.3.7. **Genus *Indolachnus* gen. nov.**

**Type species**: *Indolachnus himalayensis* (van der Goot, 1917) **comb**. **nov**., here designated.

**Etymology**. The name of the genus is derived from India, from where *N. himalayensis* was described and the word “*lachnus*”—a suffix very often used to name genera in Lachninae. Gender masculine.

**Diagnosis**. The new genus can be recognised from all species of the genus *Nippolachnus* first of all by its usually larger size of 3.45–5.50 mm (not more than 3.62 in *Nippolachnus*), pear-shaped body of apterous viviparous females (narrow oval in *Nippolachnus*), all legs uniformly black (legs at most yellow with dusky distal part of hind tibiae in *Nippolachnus*), PT with one subapical seta (PT without subapical setae in *Nippolachnus*), and mesosternal furca with robust base with large stem (mesosternal furca wide, fused very slightly with poorly developed base without stem). *Alate viviparous females* can be distinguished by numerous (over 40) small secondary rhinaria on ANT III in more than one row (not more than 10 large secondary rhinaria, usually in one row on ANT III in *Nippolachnus*), twice-branched media of fore wings (unbranched or one-branched in *Nippolachnus*), and completely membranous abdominal cuticle (abdomen with at least small central sclerite, usually with complicated pattern of sclerotisation in *Nippolachnus*).

**Description.**

*Apterous viviparous females.* Body broadly oval or pear-shaped, very densely covered with brown pigmented, long, fine, pointed setae. Head sclerotised, brown with well-developed epicranial suture. Compound eyes large without ocular tubercle (triommatidium probably under the eyes but not visible in mounted specimens). Antennae six-segmented. ANT IV with 1–2 small, rounded secondary rhinaria on the apical part. Antennal setae extremely numerous, very fine, pointed, longer than the width of the segments. Primary rhinaria oval without sclerotised rim. PT well developed, distinct, with one subapical seta and without long, fine setae. Accessory rhinaria on BASE near the major rhinarium, only one rhinarium can shift to the proximal part of the terminal process. Rostrum long, reaching hind coxae, sclerotised rostrum groove distinct, strongly sclerotised, longer than 0.80 mm. Legs completely black, very densely covered with setae, as long as or shorter than the width of tibiae. First segment of tarsi dorsal length as long as the basal length. Abdomen membranous, light brown pigmented with light brown pigmented siphuncular sclerites. Genital and anal plate distinct, brown. Cauda broadly rounded.

*Alate viviparous females.* Head and thorax sclerotised. Head with epicranial suture. Antennae with numerous moderately sized, protuberant secondary rhinaria located irregularly over the whole length of segments. Hind wings with short pterostigma and Rs bent in the middle of its length. Media twice-branched (Figure 17g). Cubital veins straight, arising separately. Abdomen membranous, without sclerotisation.

***Indolachnus himalayensis* (van der Goot, 1917) comb. nov.**
 Van der Goot, 1917 [16], p. 180
 *Nippolachnus eriobotryae* Basu and Hille Ris Lambers, 1968 [20], p. 11

(Figure 13, Figure 14, Figure 15, Figure 16, Figure 17, Figure 18 and Figure 24; Table 1)

**Apterous viviparous female**—re-description

*Colour*: in life, body brick red with very distinct segmentation, with whitish intersegmental lines. Legs blackish (Basu and Hille Ris Lambers 1968). Mounted specimens: head sclerotised, brown, antennae brown with slightly paler basal part of ANT III and darker ANT V and VI. Thorax and the rest of the body light brown, extremely densely covered with very fine, brown pigmented setae. Legs uniformly dark brown to black (Figure 13h and Figure 15h). *Morphometric characters*: HW 0.43–0.56 × ANT. ANT 0.31–0.43 × BL. ANT IV always shorter than ANT V with 1–2 secondary rhinaria. ANT V longer than ANT VI. ANT VI with 25–33 basal setae. PT without long, fine setae, with one short, thick and stiff seta (Figure 14h). ANT VI PT 0.46–0.51 × BASE. Other antennal ratios: VI:III 0.37–0.44, V:III 0.44–0.50, IV:III 0.36–0.41. LS III 2.40–2.75 × BD III. URS 0.39–0.43 × ANT III, 0.97–1.04 × ANT VI, and 0.63–0.65 × HT II with 28–31 accessory setae. Hind tibiae setae 0.06–0.15 mm long. HT I with numerous ventral setae (except sense peg). HT II 0.62–0.66 × ANT III and 1.48–1.63 ANT VI. Dorsal setae, 0.10–0.16 mm long. ABD VIII with 65–74 setae.

**Alate viviparous female**—re-description based on van der Goot [16], Basu and Hille Ris Lambers [20], and Ghosh AK [4].

*Colour*: in life, head and thorax brownish. Abdomen brick red. *Mounted specimens:* head and thorax brownish, sclerotised. Antennae brown. Abdomen pale, membranous. *Morphometric characters*: ANT about 0.39 × BL. ANT III with 44–60 small, round or oval and protuberant secondary rhinaria distributed irregularly along the segment, ANT IV shorter than ANT V with 10–11 secondary rhinaria. ANT V shorter than ANT VI with 4–7 secondary rhinaria (Figure 17g). ANT VI PT about 0.41 × BASE. Other antennal ratios: VI:III 0.39–0.42, V:III 0.42–0.50, IV:III 0.42–0.44. URS about 0.66 × HT II.

**Material examined**. Paratypes. INDIA, West Bengal, Darjeeling, 1 Jun. 1957, *Eriobotrya petiolata*, one apt. viv. fem., one nymph, A.N. Basu leg., BM 1984-340 (A); one apt. viv. fem., two nymphs, BM 1984-340 (B); one apt. viv. fem., one nymph, BM 1984-340 (C).

**Diagnosis**. Characters as in the genus diagnosis.

**Host plants**. *Eriobotrya dubia* and *E. petiolata* on which the aphid forms large colonies on the underside of leaves and petioles without injury to the plant.

**Distribution**. The species is known to date only from Darjeeling in West Bengal (India). Remarks. Ghosh AK [4] listed *Lachnus fici* as a probable synonym of this species. On the other hand, Tao [34] listed *N. himalayensis* as a synonym of *Lachnus fici*. It can be concluded from the first examination of the material and description [39] of this species that the authors have probably never seen specimens of *L. fici.*

## 4. Discussion

### 4.1. Relationships within Nippolachnus

Our phylogenetic analyses confirmed the monophyly of the genus *Nippolachnus* and its sister group relationships with the newly established genus *Indolachnus* gen. nov. The phylogenetic results support the morphological observation and showed that a separate genus should be established for *N. himalayensis*. Taking into account morphological similarities, several groups of species can be distinguished within *Nippolachnus* when analysing different morphs. In the case of apterous viviparous females, the most important differentiating character is the pigmentation of legs and antennae. In this regard, two species—*N. bengalensis* and *N. chakrabartii* sp. nov.—stand out the most by having the most pigmented hind tibiae, yellow to pale brown in *N. bengalensis* and brown with darker apices and additionally brown distal parts of femora in *N. chakrabartii* sp. nov. Moreover, *Nippolachnus bengalensis* differs from all other *Nippolachnus* species in having an antennal terminal process without long setae, a relatively low value of the PT/BASE ratio of 0.27–0.32 (0.41–0.69 in the other species), a low number of ANT VI basal setae 14–15 (16–27 in the other species), and URS accessory setae 8–10 (14–25 in the other species). *Nippolachnus chakrabartii* sp. nov., besides the very characteristic set of pigmentation of femora and tibiae, can be additionally distinguished by having the lowest LS III/BD III ratio, 3.00–3.66 (4.07–7.50 in the other *Nippolachnus species*), mostly due to the largest width of the basal articular diameter of ANT III and the HW/ANT ratio of 0.64–74 (0.44–0.64 in the other examined species). *Nippolachnus malayaensis* sp. nov. and *N. sinensis* sp. nov. may be another group of species which differ from all the others in having the palest body and appendages (sometimes with only pale brown very distal ends of tibiae and tarsi). Although morphologically *N. malayaensis* sp. nov. differs from *N. bengalensis* and *N. chakrabartii* sp. nov., they are relatively similar in some general features, which was also reflected in the phylogenetic results (Figure 1). Additionally, the two new species differ from the *micromeli*/*piri*/*xitianmushanus* group of species in having different numbers of basal (16–21), URS accessory (14–16), and ABD VIII setae (36–42), which in the *micromeli*/*piri*/*xitianmushanus* group are more numerous (20–27, 18–25, and 45–52, respectively). The last group of similar species, *micromeli*/*piri*/*xitianmushanus*, besides the already mentioned differences, is characterised by brown to dark pigmented distal parts of hind tibiae and antennal segments and their close relation in the results of the phylogenetic analyses (Figure 1). *Alate viviparous females* of *Nippolachnus*, due to the unique abdomen sclerotisation, are much easier to distinguish. The first group in this regard consists of *N. bengalensis* and *N. chakrabartii* sp. nov., in which the abdomen is very poorly sclerotised. On the phylogenetic tree, *N. chakrabartii* sp. nov. is a sister group to a clade formed by *N. bengalensis* and *N. malayaensis* sp. nov., in which the alatae are unknown, but perhaps their sclerotisation is similar to the two known ones. Within the mentioned species, *N. bengalensis* is the most distinctive with an almost membranous abdomen only with a small spinal sclerotic patch and residual marginal sclerites. Those characters are sufficient to distinguish *N. bengalensis* from the alate viviparous females of *N. chakrabartii* sp. nov. in which the spinal sclerotisation is much more developed (longer) and the marginal sclerites are bigger and clearly visible. The remaining known alate viviparous females of the *Nippolachnus* species, in contrast to *N. bengalensis* and *N. chakrabartii* sp. nov., are characterised by much more developed abdominal sclerotisation. From the remaining species—*N. micromeli*, *N. piri*, *N. sinensis* sp. nov., and *N. xitianmushanus—*the first one can be distinguished most easily by the presence of clearly visible sclerotic cross-bands on ABD VI and VII, which are absent in all other species (including even *N. bengalensis* and *N. chakrabartii* sp. nov.). From the rest of the species with the most similar sclerotisation pattern, *N. sinensis* sp. nov. can be distinguished from *N. piri* and *N. xitianmushanus* by having a less developed spinal sclerotic patch and the absence of marginal sclerites on ABD IV. This group of species with a well-developed sclerotisation pattern form one close group on the tree (Figure 1).

### 4.2. Indolachnus gen. nov. and Nippolachnus Differences

*Nippolachnus himalayensis* has been the most distinctive species within *Nippolachnus*, which is confirmed in our phylogenetic analyses by the sister group resolution between this species and all other *Nippolachnus*. It clearly differs from the other species in the pigmentation of alive and mounted specimens, body shape, and size. Additionally, detailed and comparative morphological analyses have shown further differences, which led us to establish a new genus, *Indolachnus* gen. nov., to accommodate the clearly different *N. himalayensis*. Our observations confirmed that the apterous viviparous females of the new genus are only superficially similar to representatives of *Nippolachnus*, which is not unusual between some genera of Lachninae (e.g., *Maculolachnus* Gaumont, 1920 and *Lachnus* Burmeister, 1835, *Eulachnus* Del Guercio, 1909 and *Essigella* Del Guercio, 1909, *Maculolachnus* and *Sinolachnus*). Besides the mentioned larger body size and shape, apterous viviparous females of *Indolachnus* gen. nov. are characterised by completely black legs and pigmented body setae (pale to only distally brown hind legs and pale setae in *Nippolachnus*). Further analyses of the apterae revealed additional differences such as the pedicel chaetotaxy, which in *Indolachnus* gen. nov. is extremely setose (only a few setae in *Nippolachnus*), the presence of one short and rigid seta (type II trichoid sensillum) on the last antennal segment terminal process (absent in *Nippolachnus* species), and large, robust, fused mesosternal furca based on a large stem (delicate and separated mesosternal furca without stem in other *Nippolachnus* species). Apterous viviparous females of the new genus also differ from the rest of the *Nippolachnus* species in having a large number of small campaniform sensilla on the inner side of the hind tibiae (only a few larger ones in *Nippolachnus* species) and a large number of setae on the first tarsal segment, which are sparse in *Nippolachnus*. In addition, the two genera can be distinguished by the difference in distribution of accessory rhinaria on the last antennal segment. In *Indolachnus* gen. nov., at most only one rhinarium (small multiporous placoid sensillum) is located on the terminal process and the remaining ones are near the major rhinarium on the basal part of the segment. In contrast, in apterae of the *Nippolachnus* species, the common feature of the distribution of accessory rhinaria is that almost all of them are located on the terminal process (sometimes one rhinarium left on the base), and they are in a lateral position to the major rhinarium (see Figure 6n and Figure 24). Differences are even more distinct in the alate viviparous females, which in *Indolachnus* gen. nov. are characterised by a large number of small secondary rhinaria (small multiporous placoid sensilla) distributed irregularly on the segments—up to 60 on ANT III, up to 11 on ANT IV, and up to 7 on ANT V (not more than 12 sensilla on ANT III, 3 on ANT IV, and 2 on ANT V in *Nippolachnus*, in which additionally the rhinaria are of large diameter and lie in one row). Besides the sensilla morphology, alate viviparous females of both genera differ in fore wing media branching (two branches in *Indolachnus* gen. nov. and one or no branch in *Nippolachnus*) and the dorsal abdominal sclerotisation, which is lacking in *Indolachnus* gen. nov. and in *Nippolachnus* plays an important role to determine species. We decided to create a new genus name to give a clear message about the outstanding morphology of *Indolachnus himalayensis* comb. nov. and to keep the diagnosis of *Nippolachnus* more robust. Undoubtedly both genera are close to each other due to some similarities and biology (e.g., the feeding place of the plant), and collecting fresh material of *Indolachnus* gen. nov. should confirm this hypothesis, which was formulated on the basis of morphological studies.

### 4.3. Phylogenetic Relationships in Lachninae

At the first sight, the phylogenetic relationships obtained in the analyses presented here may be far from the molecular-based ones (e.g., [1,8]). As many species of *Nippolachnus* and *Indolachnus himalayensis* are extremely difficult to collect in life due to the lack of specialists or legal regulations, it was impossible to obtain fresh material which could be analysed using molecular markers. Our main goal was to test the relationships within *Nippolachnus* and to establish the position of *N. himalayensis*, which does not fit the genus diagnosis. Our analyses did not recover the tribe Tuberolachnini as monophyletic, but this situation can be easily explained. Undoubtedly, *Nippolachnus* (and *Indolachnus* gen. nov.) are the most derived genera within Tuberolachnini and are an example of the high degree of morphological diversity in a tribe within Lachninae. *Nippolachnus* representatives are much more slender than the rest of the Tuberolachnini (including *Indolachnus* gen. nov.). Both genera are characterised by the unusual shifting of triommatidia behind the eyes; triommatidia are located on the ocular tubercle directly near the compound eyes in the rest of the Tuberolachnini and may be absent or only single in other Lachninae. All *Nippolachnus* species are pale or pale green, which is also unusual within the Lachninae, which feed above ground (only root-feeding Tramini are characterised by a whitish body), and the vast majority of which are brown to dark or reddish like *Indolachnus himalayensis* comb. nov. Moreover, the length of ANT III in relation to other segments also differs among Tuberolachnini genera—shorter in *Nippolachnus* and *Indolachnus* gen. nov. and longer in *Pyrolachnus* and *Tuberolachnus*. There is also a difference between *Nippolachnus* and *Indolachnus* gen. nov. in feeding place, which is completely different from *Pyrolachnus* and *Tuberolachnus* representatives. Most likely in both genera (*Nippolachnus* and *Indolachnus* gen. nov.), due to the change of the feeding place (leaves and additionally petioles in *I. himalayensis* comb. nov.), morphological features have undergone several changes, which have made them so distinctive. This unique morphology of *Nippolachnus*, before the molecular studies confirmed their affiliation with Tuberolachnini, was the reason why, for some time, it was treated as a separate tribe—*Nippolachnini* [40]. One more species of Tuberolachnini—*Pyrolachnus imbricatus nipponicus* Sorin, 2011—also seems to be far from the other species of *Pyrolachnus* and Tuberolachnini, but in this case, several important differences were noted and placed the generic and tribal identity of this species in question. As *P. imbricatus nipponicus* shares some features with *Sinolachnus yushanensis* Kanturski et al. [14], those taxa formed one clade; however, this needs to be confirmed by molecular study, and this result should not question *Sinolachnus* as a member of Tramini [14]. Stomaphidini and root-feeding Tramini formed sister groups, as always in morphological investigations [41], probably due to a similar life mode (underground or often deep under the bark), despite the fact that Stomaphidini are the sister group to the clade formed by Tramini and Tuberolachnini in recent molecular results [1]. The last unexpected result of the phylogenetic analyses is the position of *Lachnus tatakaensis*, which was resolved outside Lachnini, in the clade formed by Tramini, Tuberolachnini, and Stomaphidini. The species was deliberately chosen to test the hypothesis of Blackman and Eastop [10] that it is doubtfully congeneric with other *Lachnus* species. In conclusion, the poor resolution of the backbone is not a surprise since the analyses only concerned morphological data of highly derived taxa. Further studies and analyses should be conducted to thoroughly elucidate the relationships within Tuberolachnini and other tribes.

### 4.4. The status of Neonippolachnus and N. betulae Shinji, 1924

Shinji [17], in the same paper, described two taxa—*N. micromeli* from *Sorbus alnifolia* (=*Micromeles alnifolia*) and a new genus and species, *Neonippolachnus betulae* from *Betula* sp. As Shinji made his descriptions mostly from alive specimens or alcohol-preserved samples, they were always short and very general (most likely Shinji had the ability to recognise alive aphids as a new species), often without details and measurements of some discrete but relevant characters (especially in Lachninae). The *Neonippolachnus* diagnosis is therefore equally enigmatic and we can read the following: “Much resembling *Nippolachnus* Matsumura, but the cornicle is rather like that of *Eulachnus* Del Guercio in not arising from a hairy cone but directly from the side of the body, ½ as long as the diameter, no hair, as broad as the apex at the base”. On the other hand, from the short description of the alate viviparous females, some characters could probably, in fact, be of *Nippolachnus*: “antenna short, ANT III with five large circular sensoria arranged in one row, IV the shortest piece with 2 sensoria, V with one sensorium. Wings as in *Nippolachnus piri* Mats. Abdomen with black maculate on the dorsum” (Shinji 1924). Regarding the apterous viviparous female, Shinji [17] gave only one character—the live specimen colour—“light yellow, almost subhyaline”. The description of *Neonippolachnus betulae* was also the last one during almost a whole century of intensive activity of excellent Japanese aphidologists (Akimoto, Aoki, Higuchi, Hori, Miyazaki, Moritsu, Sano, Sasaki, Sorin, Yoshitomi, etc.). After Shinji’s publication, only a very few researchers took up the topic of the existence and status of the genus. The one who relatively quickly raised doubts about *Neonippolachnus* was Takahashi [42], who according to the short description was willing to place the species in *Eulachnus*. What is interesting, Shinji himself in his large publication “Nippon Aburamushi Sosetsu” [Monograph on the Japanese Aphids] [32] listed only *Nippolachnus piri* (with *N. micromeli* as a synonym) and there was no mention about *Neonippolachnus*. Later, the genus and species were mentioned only a few times, in, e.g., catalogues of aphids [18,19,43,44], without further comments on its status. Recently, *Neonippolachnus* has also been mentioned by Chen et al. [1] as a possible synonym of *Nippolachnus*, referring to Blackman and Eastop [10]. The latter in fact put the information on the possible status of *Neonippolachnus* in the *N. piri* section after the discussion with the first author during his internship in the Natural History Museum in London (UK).

As we mentioned at the beginning of this section, the description of *Neonippolachnus* is rather poor but we can indicate some characters mentioned by Shinji (1924), which confirm the synonymy of *Neonippolachnus* with *Nippolachnus*. First of all, Shinji [17] indicated that representatives of the new genus “much resemble” *Nippolachnus* and confirmed it in the description of the antennae (large sensoria in one row and ANT IV with two sensoria) and wings (as in *Nippolachnus*). Of course, Shinji [17] indicated that the siphunculi are without setae and not placed on cones, but taking into account observations of al. viv. fem. in life (especially those freshly moulted and without wax secretion) (e.g., Figure 2d), it is difficult to say that their siphunculi are placed on elevated hairy cones. Large, circular sensoria are one of the most important features distinguishing *Nippolachnus* from other Tuberolachnini and Lachninae as a whole, especially ANT IV, which almost always bear two secondary rhinaria in alatae (especially *N. micromeli* and *N. piri*). A feature which additionally matches the fundamental characters of al. viv. fem. of *Nippolachnus* is a central abdominal patch, composed of differently developed dorsal abdominal sclerotisation on especially ABD III–V. This character clearly distinguishes *Nippolachnus* representatives from other Tuberolachnini and Lachninae, including *Eulachnus* (from which Shinji provided the diagnosis of *Neonippolachnus*). The mentioned features do not in any case correspond to Calaphidinae genera, which can feed on *Betula*, such as *Betulaphis* Glendenning, 1926, *Calaphis* Walsh, 1863, *Euceraphis* Walker, 1870 or *Callipterinella* van der Goot, 1913. Although, e.g., *Calaphis neobetulella* Quednau, 1971 alatae are characterised by a dark central patch on the abdomen, their antennae are evidently as long as or longer than the body. The last argument that confirms the synonymy of *Neonippolachnus* with *Nippolachnus* is the colour of apterous viviparous females (or alatoid nymph), which have been described as “light yellow, almost subhyaline” [17], which matches the colour of live specimens of *N. micromeli* and mostly not green *N. piri*, see [13]. The last argument concerns the host plant—*Betula* sp. Shinji [17] did not give any further information on the species. Of course, we can consider that some al. viv. fem. flew over from their host plants and they were there accidentally (they could even give birth to the apterous viviparae). On the other hand, if Shinji was not able to determine the species, it is not impossible that he collected the specimens from Betula grossa, which is similar to *Sorbus alnifolia* (host plant of *N. micromeli*) and could be simply confused.

## 5. Conclusions

This study showed that despite a lack of material for molecular analyses, detailed and comparative morphological analyses with a phylogenetic approach can be helpful and useful for solving taxonomical problems in poorly known aphid taxa. Although morphological analyses with a phylogenetic approach helped to solve taxonomical problems in this study, undoubtedly in the future, combining morphological and molecular data will be more convincing. More work needs to be done to clarify further issues within the Tuberolachnini as well as other tribes within the Lachninae, which is the key interest of the first author.

We propose the following genera to be members of the tribe Tuberolachnini: *Indolachnus* gen. nov., *Nippolachnus*, *Pyrolachnus*, and *Tuberolachnus*.

## Data Availability

All data generated or analysed during this study are included in this published article.

## Supplementary Materials

The following supporting information can be downloaded at: https://www.mdpi.com/article/10.3390/insects15030182/s1.
