# Supplementary figures and images for: Taxonomy and Phylogeny of the Aphid Genus Nippolachnus Matsumura, 1917, with Synonymy of the Mysterious Neonippolachnus Shinji, 1924 (Hemiptera: Aphididae: Lachninae)
Source: Insects. 2024 Mar 8;15(3):182. doi: 10.3390/insects15030182 (PMC10971630; doi:10.3390/insects15030182)

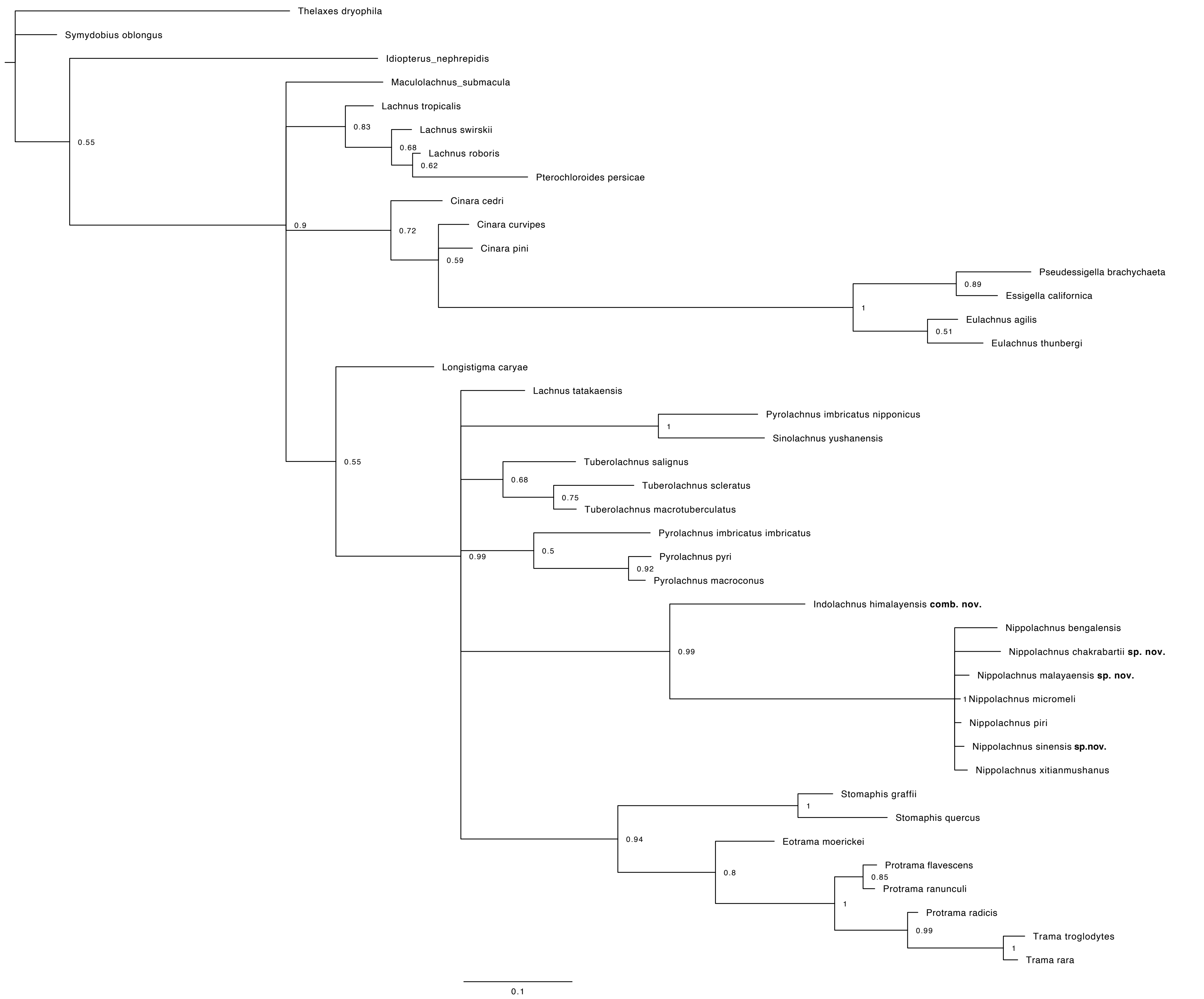

Supplement: Supplementary file 1 [file insects-15-00182-s001.zip › File_4.pdf]
